# Climate change and variability impacts on grazing herds: Insights from a system dynamics approach for semi‐arid Australian rangelands

**DOI:** 10.1111/gcb.14669

**Published:** 2019-06-24

**Authors:** Cecile Godde, Kanar Dizyee, Andrew Ash, Philip Thornton, Lindsey Sloat, Eugeni Roura, Benjamin Henderson, Mario Herrero

**Affiliations:** ^1^ Agriculture and Food Commonwealth Scientific and Industrial Research Organisation St Lucia Australia; ^2^ The University of Queensland St Lucia Australia; ^3^ CCAFS, International Livestock Research Institute Nairobi Kenya; ^4^ University of California Irvine Irvine California; ^5^ Organisation for Economic Co-operation and Development Paris France

**Keywords:** climate change, climate variability, grasslands, greenhouse gas emissions, intensification, livestock modelling, system dynamics, vulnerability

## Abstract

Grazing livestock are an important source of food and income for millions of people worldwide. Changes in mean climate and increasing climate variability are affecting grasslands' carrying capacity, thus threatening the livelihood of millions of people as well as the health of grassland ecosystems. Compared with cropping systems, relatively little is known about the impact of such climatic changes on grasslands and livestock productivity and the adaptation responses available to farmers. In this study, we analysed the relationship between changes in mean precipitation, precipitation variability, farming practices and grazing cattle using a system dynamics approach for a semi‐arid Australian rangeland system. We found that forage production and animal stocking rates were significantly affected by drought intensities and durations as well as by long‐term climate trends. After a drought event, herd size recovery times ranged from years to decades in the absence of proactive restocking through animal purchases. Decreases in the annual precipitation means or increases in the interannual (year‐to‐year) and intra‐annual (month‐to‐month) precipitation variability, all reduced herd sizes. The contribution of farming practices versus climate effect on herd dynamics varied depending on the herd characteristics considered. Climate contributed the most to the variance in stocking rates, followed by forage productivity levels and feeding supplementation practices (with or without urea and molasses). While intensification strategies and favourable climates increased long‐term herd sizes, they also resulted in larger reductions in animal numbers during droughts and raised total enteric methane emissions. In the face of future climate trends, the grazing sector will need to increase its adaptability. Understanding which farming strategies can be beneficial, where, and when, as well as the enabling mechanisms required to implement them, will be critical for effectively improving rangelands and the livelihoods of pastoralists worldwide.

## 1 INTRODUCTION

Climate change and variability is a major concern for grazing systems worldwide. Projected increases in the frequency and severity of extreme climate events (e.g. heat stress, drought and flooding) as well as drier conditions in part of the world, especially in arid and semi‐arid regions (Herrero et al., 2016; Kitoh & Endo, 2016; Warszawski et al., 2014), are expected to have significant negative consequences on herd populations as a result of decreases in feed and water quantity and quality, declined reproductive performance, heat stress, and increased disease incidence and mortality (as reviewed in Rojas‐Downing, Nejadhashemi, Harrigan, & Woznicki, 2017; ThorntonSteeg, Notenbaert, & Herrero, 2009). These reductions in animal numbers threaten local livelihoods, especially in regions that are dependent on livestock as a source of food or income. Additional benefits that livestock can confer, such as non‐food products and capital functions, may also be compromised and the conflicts over animal assets present in some regions may escalate, especially in Africa (e.g. Horn of Africa). Climate change may result in reduced land carrying capacity and associated overgrazing, which leads to losses in ecosystem goods and services. Some regions in high latitudes may, however, not suffer these negative impacts, with pasture and livestock productivities potentially increasing due to more suitable temperatures (Herrero et al., 2016).

In general, inter‐ (i.e. year‐to‐year) and intra‐annual (i.e. within a year, month‐to‐month) precipitation variability are key drivers of forage production (e.g. field studies: Bat‐Oyun, Shinoda, Cheng, & Purevdorj, 2016; Craine et al., 2012; Le Houérou, Bingham, & Skerbek, 1988; Yang, Fang, Ma, & Wang, 2008; modelling studies: Guan et al., 2014; Peng et al., 2013; Sloat et al., 2018). Some studies have also reported that precipitation was a proxy predictor of livestock population dynamics in extensive pastoral systems where long‐term data series were used in the analysis (e.g. household surveys: Angassa & Oba, 2013, 2007; modelling studies: Hahn et al., 2005; Lunde & Lindtjørn, 2013). An increased incidence of droughts, related to changes in precipitation variability, may also affect forage production and herd size e.g. surveys: Angassa & Oba, 2013; Desta & Coppock, 2002; Homewood & Lewis, 1987; McCabe, 1987; Oba, 2001; farm experiments: O'Reagain & Bushell, 2011; modelling study: Hatch & Stafford Smith, 1997).

Over the last century, inter‐ and intra‐annual precipitation variability have generally increased across global grasslands (Sloat et al., 2018), although both positive and negative trends exist. The rates of year‐to‐year variability increase appear to be largest in regions where livestock grazing is important for local food access and/or economies (e.g. Sahel, Somalia, Kenya, Zimbabwe, Australia; Sloat et al., 2018). Moreover, changes in climate variability may counterbalance the impacts of changes in mean variables alone (IPCC, 2012).

Despite the growing economic, social and environmental threats associated with such precipitation changes, climate change and variability impacts on short‐ and long‐term herd dynamics have been understudied (Thornton, Ericksen, Herrero, & Challinor, 2014; Thornton et al., 2009). Field research often focuses on vegetation dynamics. When they do consider animal herds, these studies usually provide information related to specific management and weather patterns of relatively short duration, and do not necessarily detail climate mean and variability characteristics nor consider management effects. Furthermore, most studies focus on average herd variables such as growth and reproduction, and not on the temporal changes in herd dynamics including animal numbers (e.g. 1‐ to 2‐year survey data analyses: Homewood & Lewis, 1987; Oba, 2001; 5 years: McCabe, 1987; 15 years: Angassa & Oba, 2013; 17 years: Desta & Coppock, 2002).

Modelling studies, while being a simplified representation of actual systems, can provide additional insights by allowing impact analyses of a wide range of farming practices and short‐ and long‐term climate scenarios. The level of details in herd‐forage models to capture climate change effects vary. For example, some models do not represent the direct feedback of animal forage intake on forage availability (e.g. Hatch & Stafford Smith, 1997; Pulina, Salimei, Masala, & Sikosana, 1999). Some models run on an annual basis, thus do not allow the capture of seasonal climate variability effects (e.g. Bénié, Kaboré, Goïta, & Courel, 2005; Beukes, Cowling, & Higgins, 2002; Hahn et al., 2005; Hatch & Stafford Smith, 1997; Janssen, Walker, Langridge, & Abel, 2000; Perrings & Walker, 1997; Wu, Li, Stoker, & Li, 1996). Detailed models that run on a monthly or weekly time step have also been developed (e.g. models reviewed in Bryant & Snow, 2008). However, these models are complex in their pasture and animal dynamics and are constrained in their ability to assess the specific effects of a wide range of climate scenarios. In this study, we aim to provide novel insights into the potential impacts of climate change and variability on rangeland production systems by developing a purpose‐built system dynamics model that allows the impacts of a wide range of climate scenarios on long‐term herd dynamics to be assessed. We also study the influence of intensification strategies and implications for enteric methane emissions. This study takes a case‐study approach in a semi‐arid Australian rangeland system, system constrained by high and increasing climate variability. We hypothesize that such an environment could be significantly impacted by a changing climate.

## 2 METHODS

### 2.1 Framework

**Figure 1:**
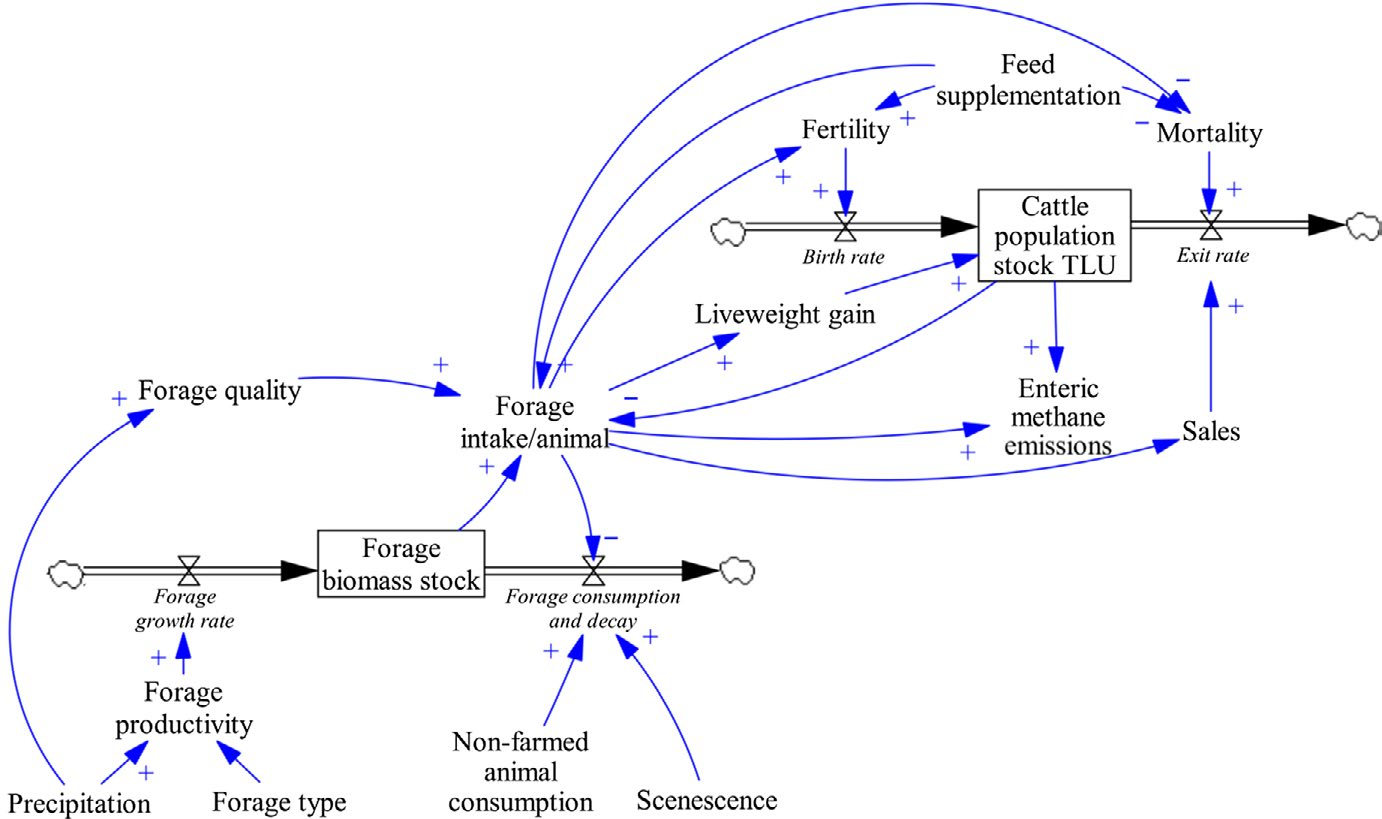
Simplified representation of key stocks, flows and causal linkages in the model. The model portrait includes stocks in which resources accumulate at a particular point in time (boxes) and flows which compute the rate of change into and out of the stock (thick arrows with valves). The rate at which flows enter or exit stocks as well as other technical relationships that indirectly affect this rate are influenced by parameters, also called converters. A positive (+) sign on the arrowheads indicates that a change in a source variable will change the destination variable in the same direction (e.g. an increase in animal sales contributes to a decreased herd stock). In contrast, a negative (−) sign indicates that the variables move in opposite directions (e.g. an increase in forage intake per animal contributes to a decrease in the forage biomass stock and vice versa). The figure was designed with Vensim software (Ventana Systems, 2017). A detailed description of the herd and forage components of the model, as well as underlying differential equations, are presented in Appendix S1)

To explore the dynamic behaviour of cattle herds under changing climate and farming scenarios, we constructed an integrated forage and herd model that links precipitation regimes to forage production, quality and herd dynamics (e.g. animal forage intake, liveweight gain, fertility and mortality rates) on a weekly basis (Figure 1).

We assessed the short‐ and long‐term impacts (up to 30 years) of a wide range of climate scenarios on forage and herd dynamics. We also assessed the impact of farming intensification strategies that focussed on pasture improvement to increase forage production and carrying capacity, and animal feed supplementation. The production impacts of intensification strategies under climate scenarios also provide information as to their potential as a climate adaptation strategy. Climate and management impacts on enteric methane emissions were also assessed. These production, adaptation and mitigation considerations under a wide range of climate scenarios aimed at providing novel insights to key pillars of the climate‐smart agriculture and United Nations Sustainable Development Goals frameworks (Lipper et al., 2014; United Nations, 2015).

Economic analyses were not the focus of this modelling study, which represents a subset of a beef enterprise and does not include farm revenues or costs. The herd was managed to ensure pasture utilization rates that are considered for the region as economically and environmentally sustainable and limit herd mortalities (~20%; Ash, Corfield, McIvor, & Ksiksi, 2011; Hunt, 2008; O'Reagain & Bushell, 2011; O'Reagain, Scanlan, Hunt, Cowley, & Walsh, 2014) rather than in a specific attempt to maximize profits. Farming practices modelled (pasture sowing and low‐cost per head feed supplementation) were within the range observed in the region (McIvor & Gardener, 1995; McIvor & Monypenny, 1995; McLennan, Hirst, Shepherd, & McGuigan, 1991; Peck et al., 2011; Walker & Weston, 1990). Herd size rebuild after droughts was modelled through herd management (e.g. males were sold in priority as compared to females during droughts; young females were retained in the herd during the herd recovery phase). This allowed us to explore herd recovery periods in the absence of proactive rebuild through animal purchases. In practice, pastoralists may try to rebuild their herd through animal purchases as this can restore profitability more quickly (Buxton & Smith, 1996). However, this option can be challenging due to increased cattle scarcity and associated high cattle prices after a widespread drought (Hatfield‐dodds, Hughes, Cameron, Miller, & Jackson, 2018).

### 2.2 Case study

**Figure 2:**
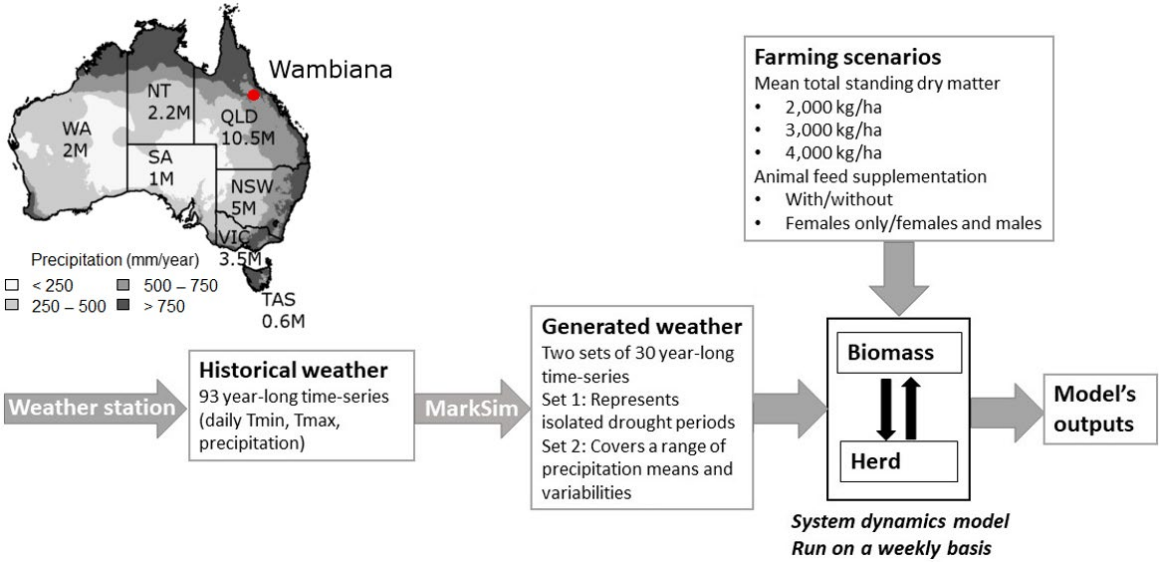
Location of the Wambiana case study in Australia, states cattle numbers and study framework. Climate data from Jones, Wang, and Fawcett (2009) (mean over years 1981–2012). Cattle numbers in million head (M) from the Australian Bureau of Statistics (2016)

A cattle operation (Wambiana Station) in northern Queensland, Australia (20.554666°S, 146.110317°E, Figure 2) was chosen as the case‐study site for this modelling assessment, due to the availability of long‐term historical forage biomass and stocking rates data as well as herd characteristics and stock management records, which were used to structure and parameterize the model. This region is also of interest as it has a high interannual precipitation variability (CVP‐inter = 0.37) and a high climate seasonality with 80% of rain occurring between November and April (Bureau of Meteorology, 2018; Sloat et al., 2018), resulting in herds being sensitive to climate patterns. Increases or decreases in climate variability in this region could have significant implications for livestock production and its interaction with land condition.

The meat production system modelled in this study is the one dominant in central and northern coastal Queensland, accounting for around 30% of the Australian herd (and 65% of the Queensland cattle; Meat & Livestock Australia, 2017). In this region, young males are castrated at 6 months, and steers are sold at an average liveweight of around 550 kg/head (2–3 years old). Females, when in excess of the requirement to replace breeding animals, are sold at 1–2 years old, whereas breeding animals are kept until about 9 years old. Bulls are either bred on farm or purchased at 2–3 years of age and are usually kept for around 5 years as this is considered their optimal productive lifespan for breeding.

The land used for beef operations in Australia is either freehold or leasehold, with leasehold dominating the more variable rangeland regions. Leasehold tenure is usually for many decades with conditions that are not onerous which, in effect, confers private ownership of pastoral operations. Properties are usually family or company owned and can be held for many generations within family structures. Given this security of tenure, most operations aim for long‐term sustainability, although the challenges in matching forage supply and forage demand in a highly variable climate with frequent droughts can lead to cycles of degradation and recovery. Total standing dry matter (TSDM) forage levels for the Wambiana region are around 2,000 kg/ha, and if conservative grazing management is practised, moderate stocking rates are around 0.19–0.24 tropical livestock units (1 TLU = 250 kg) per hectare and corresponding safe pasture utilization rates (percentage of annual forage growth consumed by the herd) are around 20% (O'Reagain & Bushell, 2011). This conservative management, which aims at minimizing the risks of exhausting the forage resource, is not universally adopted within the region, where higher long‐term stocking rates can be used, and contrasts with many grazing systems around the world where animals graze on communal land.

### 2.3 Forage and herd model

The herd‐forage model was developed using a system dynamics modelling approach and was run on a weekly basis over 30 years under different climate and farming scenarios (Figure 2). A lead‐in period of 60 years was used for each climate and farming practice combination to allow the model's stocking rates to stabilize in their spread of values: under very favourable climatic conditions, it takes some years for the stock numbers to increase from their initial stocking rate of 0.2 TLU/ha, as an input in the model, to values around 0.6–0.9 TLU/ha through natural replacement with no purchases, as for climate scenario S 15 (Figure 5). The section below presents some of the key model components. Further details, including the model underlying differential equations, are provided in Appendix S1.

We considered forage production and intra‐annual variations in forage quality as endogenous in the herd model, based on precipitation, to allow a direct and transparent representation of the feedback of animal consumption on forage availability (Figure 1).

A system dynamics approach is well suited to represent complex structures such as forage–herd interactions that include long feedback cycles between climate, forage production, animal reproduction, and growth and land condition—and often non‐linear relations among herd and biomass components. The approach helps framing, understanding and discussing the complex issues by assessing key behaviours over time and facilitating the evaluation of constraints and leverage points (Forrester, 1961; Sterman, 2011, 2002). In system dynamics models, profiling the evolution of the process has priority over finding a specific equilibrium or optimal solution. The basis of the method is that the complex relationships among the components of the system are just as important in determining the behaviour of the system as the individual component themselves. System dynamics has been recognized as an efficient method to represent animal population dynamics (e.g. Dahlanuddin, Henderson, Dizyee, Hermansyah, & Ash, 2017; Dizyee, Baker, & Rich, 2017; McRoberts, Nicholson, Blake, Tucker, & Padilla, 2013; Naziri, Rich, & Bennett, 2015; Rich, 2007; Rich et al., 2017; Rich & Roland‐Holst, 2014; Stephens et al., 2012).

The dynamics in the model were captured by a series of stocks (e.g. herd or standing forage biomass stocks) and flows (e.g. birth rates or forage consumption rates over time) and their changing relationships and behaviours through time were modelled using integral and non‐linear differential calculus (see Figure 1 for a simplified representation of the model key dynamics). Forage availability and quality as well as herd fertility, liveweight gains, mortality and sales rates determine the size of these flows, and therefore, the size of the cattle stock at any given point in time. When the model is in equilibrium, the inflows (births) and outflows (deaths and sales) are equal and the population is steady. The model was programmed using Stella Architect software v1.5.1 (isee systems, 2017).

#### 2.3.1 Forage model component

##### Forage biomass availability

The forage biomass stock is equal to the forage growth minus farmed cattle and wild animals' consumption and biomass senescence. We developed a simplified biomass growth model. Precipitation has a 6 week lagged logarithmic‐shaped effect on biomass growth (lag effect mentioned, e.g. in Bat‐Oyun et al., 2016; Moran et al., 2014). Similar to the pasture growth model GRASP, weekly senescence rates are larger in December to represent detachment rate for carryover material, including the impact of storms (McKeon, Ash, Hall, & Stafford Smith, 2000).

##### Forage biomass quality

Intra‐annual variations in forage quality were taken into account by estimating seasonal variations in voluntary food intake based on the approach used in producing Australia's National Greenhouse Gas Inventory (Commonwealth of Australia, 2016a).

In addition, cattle liveweight gain increases by 20% in summer, autumn and winter (December–August) if the annual number of growing weeks (precipitation >10 mm/week; McCown, Gillard, Winks, & Williams, 1981) is more than 13 weeks. If it is less than 13 weeks, no additional effect on liveweight gain was modelled as forage availability is the key limiting factor compared to forage quality. These rules contributed to represent the fact that precipitation distributed over the year was more favourable to grass quality over the year than precipitation regimes concentrated over a very limited number of weeks.

#### 2.3.2 Herd model component

##### Herd structure

The cattle herd population was comprised of interlinked animal cohorts, grouped based on their age, purpose and gender (Table 1). The grouping followed the herd categories provided in Australia's National Greenhouse Gas Inventory (Commonwealth of Australia, 2016a), which included liveweight, liveweight gain and voluntary intake estimates for each of these cohort categories. The liveweight gain estimates have an impact on the rate at which males move between cohorts. In our model, bulls are considered as constant in numbers: farmers usually keep them on the property for around 5 years and try to maintain a relatively constant bull to female ratio (usually 3%–4%; McGowan et al., 2014). Key model characteristics are described in Table 1.

**Table 1:** Some model characteristics

Variable	Value	Reference
Annual fertility rate	Max. 75% (maximum value)	McGowan et al. (2014)—northern forest region (includes Wambiana)
Gestation time	39 weeks	Konandreas and Anderson (1982)
Gender probability at birth	0.5	Konandreas and Anderson (1982)
Calving interval	1 year	Hunt et al. (2014)—seasonal mating assumption—northern beef industry
Age at first calving	2–3 yo	O'Reagain and Bushell (2011)
Annual abortion rate	8%	McGowan et al. (2014)—northern forest region (includes Wambiana—foetal/calf loss = 14%)
Annual mortality rate	Calves (0–0.5 yo): 6% Others (>0.5 yo): 3% (minimum value)	McGowan et al. (2014)—northern forest region (includes Wambiana)—foetal/calf loss = 14% Hunt et al. (2014)—assumptions for Charters Towers case study, near Wambiana
Average annual animal liveweight for the different cohorts in the model (vary by seasons in the model)	Calves (0–0.5 yo): 156 kg Steer (0.5–1 yo): 162 kg Steer (1–2 yo): 323 kg Steer (2–3 yo): 474 kg Steer (3+ yo): 567 kg Bull (1+ yo): 680 kg Female (0.5–1 yo): 153 kg Female (1–2 yo): 288 kg Female (2–3 yo): 416 kg Female (3+ yo): 459 kg	National Inventory (Commonwealth of Australia, 2016a)—average weight for Queensland, moderate/low region (includes Wambiana)

Abbreviation: yo, years old.

##### Animal forage intake estimate

###### Voluntary food intake

We used an Australia‐specific method based on cattle liveweight and liveweight gain (Minson & McDonald, 1987) to estimate potential voluntary food intake (kg DM/TLU/week). Intra‐annual seasonal variations in forage quality affect the voluntary food intake values. Additional effects of variations in forage quality related to precipitation distribution patterns are represented in the model as directly influencing liveweight gains. Additional intake by lactating cows for milk production was accounted for (SCA, 1990). The voluntary food intake equation and region‐ and season‐specific liveweight, liveweight gain and lactation feed adjustment estimates for the different cattle age and gender categories of our model can be found in the Australian National Inventory Report (Commonwealth of Australia, 2016b).

###### Actual food intake

The actual food intake per animal is lower than the potential voluntary food intake when forage availability is low and animal competition for feed is high. We also account for the fact that the higher the competition for feed among animals, the less non‐palatable parts are left ungrazed. The actual food intake influences forage availability and herd fertility, mortality, liveweight gains as well as emergency drought selling rules.

##### Breeding season

We assume seasonal mating. Bulls are allowed to mate with cows over a 4 month period, from the beginning of December until the end of March. Indeed, farmers aim to have calves from September to December, the period of the year when forage availability and quality is usually at its best (Rudder & Mccamley, 1972; Sutherland, 1961).

##### Steer liveweight gain

Steer liveweight gain depends on the actual food intake estimate. This liveweight gain–intake relationship was developed from estimates of average liveweight, liveweight gains and voluntary intake for the different animal cohorts (Commonwealth of Australia, 2016b). Liveweight gain also depends on variations in forage quality and on the feed supplementation strategy.

##### Fertility

Fertility rate depends on the amount of forage available for intake per animal (sigmoid‐shaped relationship). When forage is not limited, the maximum fertility rate is 75% (Table 1) (McGowan et al., 2014). In this model, feed supplementation strategies prevent the fertility rate dropping below 50%. Once fertility rates drop below 50%, it becomes difficult to maintain a self‐replacing breeding herd so this threshold was chosen.

##### Mortality

The model allows for ‘normal’ losses caused by a complex set of factors not directly related to nutritional status (Table 1). For animals over 0.5 years old (i.e. not milk‐fed), these mortality rates increase with increases in nutritional stress. The implementation of feed supplementation suppresses this effect.

##### Herd sales

###### Conventional sales

In the model, steers were sold at an average liveweight of 567 kg/head (~3 years old). Females, when in excess of the requirement to replace breeding animals, were sold at 1–2 years old. Breeding females were kept until 9 years old. However, sales may happen earlier in the stage of life of the animals, as described below.

###### Sales to meet the desired stocking rate target

A desired stocking rate is indicated in the model and varies depending on the annual pasture utilization rate, the latest being defined as the percentage of annual pasture growth consumed by the herd. This desired stocking rate represents the fact that famers usually lower their stocking rate when long‐term utilization rates are over 20% as they wish to prevent forage resource exhaustion and medium to long‐term land degradation (Ash et al., 2011; Hunt, 2008; O'Reagain & Bushell, 2011; O'Reagain et al., 2014). If the actual cattle stocking rate is larger than the desired stocking rate, then a proportion of 1‐ to 2‐year‐old females is sold. The relationship between the stocking gap (actual cattle number minus desired number) and the proportion of females sold follows a square root curve shape.

###### Drought emergency sales

If forage resources available for grazing are limited, a proportion of the animals older than 0.5 year old are sold. Males are sold in priority as compared to females, which are usually retained for as long as possible to maintain a viable reproductive herd.

##### Enteric methane emissions

We estimated methane emissions from grazing cattle enteric fermentation (excluding calves) based on Charmley et al. (2015) study who reported a close relationship between dry matter intake and methane production. This relationship was derived from an analysis of Australian data of dairy and beef cattle fed diets of over 70% forage. We considered methane from manure in grazing systems as negligible due to aerobic conditions (Commonwealth of Australia, 2016a). Biogenic methane emissions were expressed as CO_2_‐eq using the 100‐year Global Warming Potential value 34 from the IPCC Fifth Assessment Report and include climate carbon feedbacks, feedbacks which measure the indirect effects of changes in natural carbon reservoirs (e.g. ocean, atmosphere) due to changes in climate (IPCC, 2014).

#### 2.3.3 Model evaluation

**Figure 3:**
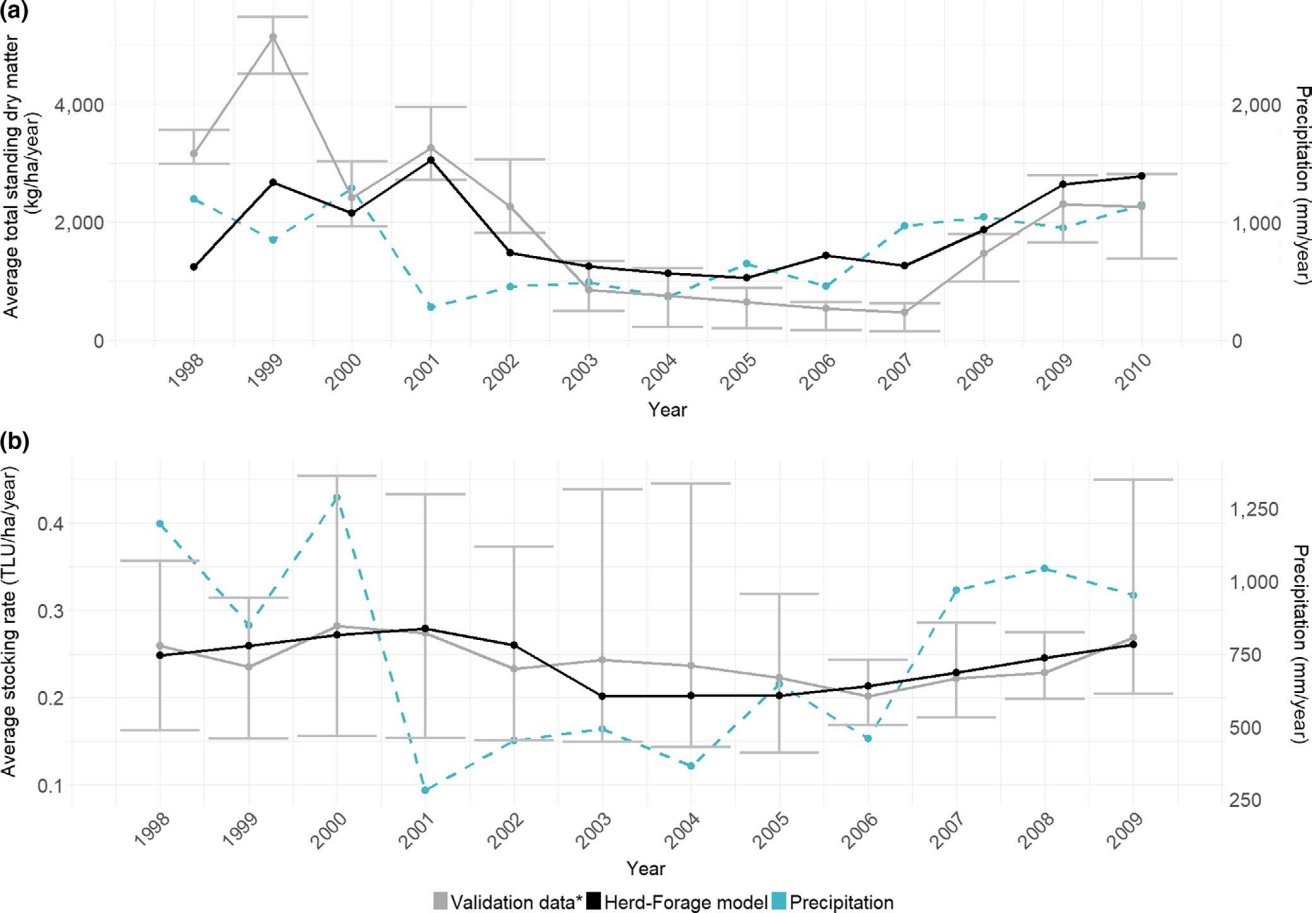
Average annual total standing dry matter (a) and stocking rates (b) over time predicted by the herd‐forage model as compared to evaluation data sets. Period: 1998–2009/2010. The error bars represent the minimum and maximum values from the evaluation data sets. *Evaluation data for total standing dry matter: Scanlan et al. (2013)—GRASP model under moderate stocking rate; O'Reagain and Bushell (2011)—heavy stocking, moderate stocking, variable stocking, rotational wet season spelling coupled with moderate‐heavy stocking, Southern Oscillation Index stocking. Evaluation data for stocking rates: Ash et al. (2015)—baseline using GRASP model; Scanlan et al. (2013)—GRASP model under moderate stocking rate; O'Reagain and Bushell (2011)—heavy stocking, moderate stocking, variable stocking, rotational wet season spelling coupled with moderate‐heavy stocking, Southern Oscillation Index stocking from a grazing experiment. TLU, tropical livestock units

Key forage and herd outputs of the model were evaluated by comparing the results from a baseline model simulation with a set of measured data for Wambiana and northern Queensland (see Figure 3 and Appendix S1 for evaluation results, Hunt et al., 2014; McGowan et al., 2014; O'Reagain & Bushell, 2011). Due to the limited amount of long‐term forage and herd measurements available in the literature, we also compared our model outputs with the ones from the GRASP model, which has been extensively used for northern Queensland including Wambiana (Ash et al., 2015; McKeon et al., 2000; Scanlan, Macleod, & O'Reagain, 2013). Key outputs included temporal variations in forage growth, TSDM and stocking rates, as well as mean stocking rates, TSDM, fertility, mortality, calving and weaning rates, forage utilization rates, forage intake as a function of forage availability, animal liveweight gains and total methane emissions over the relevant time periods. The results showed agreement between the herd‐forage model and the evaluation data sets for this climatically highly variable region. This gives confidence that the model adequately simulated these production systems.

### 2.4 Farming scenarios

The farming scenarios tested in this study were different forage production levels and animal feed supplementation strategies (Figure 2).

#### 2.4.1 Forage production level

Three levels of forage production (TSDM) were tested: (a) 2,000 kg/ha (default Wambiana productivity value for native pasture), (b) 3,000 kg/ha and (c) 4,000 kg/ha. The two higher levels of production, representing intensification strategies, are within the range observed for improved pastures grown on a low fertility soil in the region (McIvor & Gardener, 1995; Peck et al., 2011; Walker & Weston, 1990). The improved pastures are dominated by introduced grasses but can include oversown legumes and/or application of fertilizer. However, for these simulations, it was assumed that pasture quality remained constant across the different pasture productivities. These three forage production types, the values of which correspond to averages under historical climate baseline, were not fixed over time: TSDM fluctuated depending on weather, forage and herd dynamics.

#### 2.4.2 Feed supplementation

Tropical pastures across the world are usually of low quality in the dry season (i.e. low protein content and digestibility), especially so in Australia due to nutrient‐poor soils. The seasonal pattern of rainfall where more than 80% of annual rainfall falls over a few months throughout the year is also key as much forage can be available but of poor nutritional quality during the long dry season. To mitigate the impacts of low forage quality, Australian farmers often provide cattle (especially females) with urea‐type supplements, which have a high crude protein equivalent. They may also add, in fewer cases, molasses to the meal mix for its high energy content and to increase the palatability of the urea (McIvor & Gardener, 1995). To represent the impact of such intensification practices, three feed supplementation scenarios were tested: (a) no supplementation, (b) both females and males were supplemented during autumn and spring (March–November) and (c) only females were supplemented during autumn and spring. The feed supplementation effects considered were those of a combination of urea and molasses. These modest crude protein and energy supplements were provided to reduce mortality and minimize declines in female fertility and forage intake (Figure 1).

### 2.5 Climate scenarios

We generated two sets of climate scenarios based on historical weather data, to provide insights from both isolated drought events (Set 1) and long‐term trends in precipitation mean and variability (Set 2; Figure 2). Detailed precipitation characteristics of these scenarios are available in Appendix S2.

#### 2.5.1 Set 1—Drought period effect on herd stocking rate reduction and recovery time

The first set of climate scenarios represented different drought intensities and durations. We used the MarkSim weather generator (CIAT, 2001; Jones, Thornton, Díaz, & Wilkens, 2002; Thornton et al., 2002, 2014) to produce 1,000 years of weekly precipitation data based on historical daily precipitation and temperature data from Charters Towers Post Office (1900–1992), 55 km north of Wambiana cattle station (Bureau of Meteorology, 2018; Table 2).

**Table 2:** Characteristics of the historical weather data of Charters Towers Post Office, Queensland, Australia (station number 34,002, 1900–1992; Bureau of Meteorology, 2018)

Variable	Value
Mean annual precipitation (mm)	653
Standard deviation—annual precipitation	241
Interannual coefficient of variation of precipitation (CVP‐inter)	0.37
Intra‐annual coefficient of variation of precipitation (CVP‐intra)	0.87

**Figure 4:**
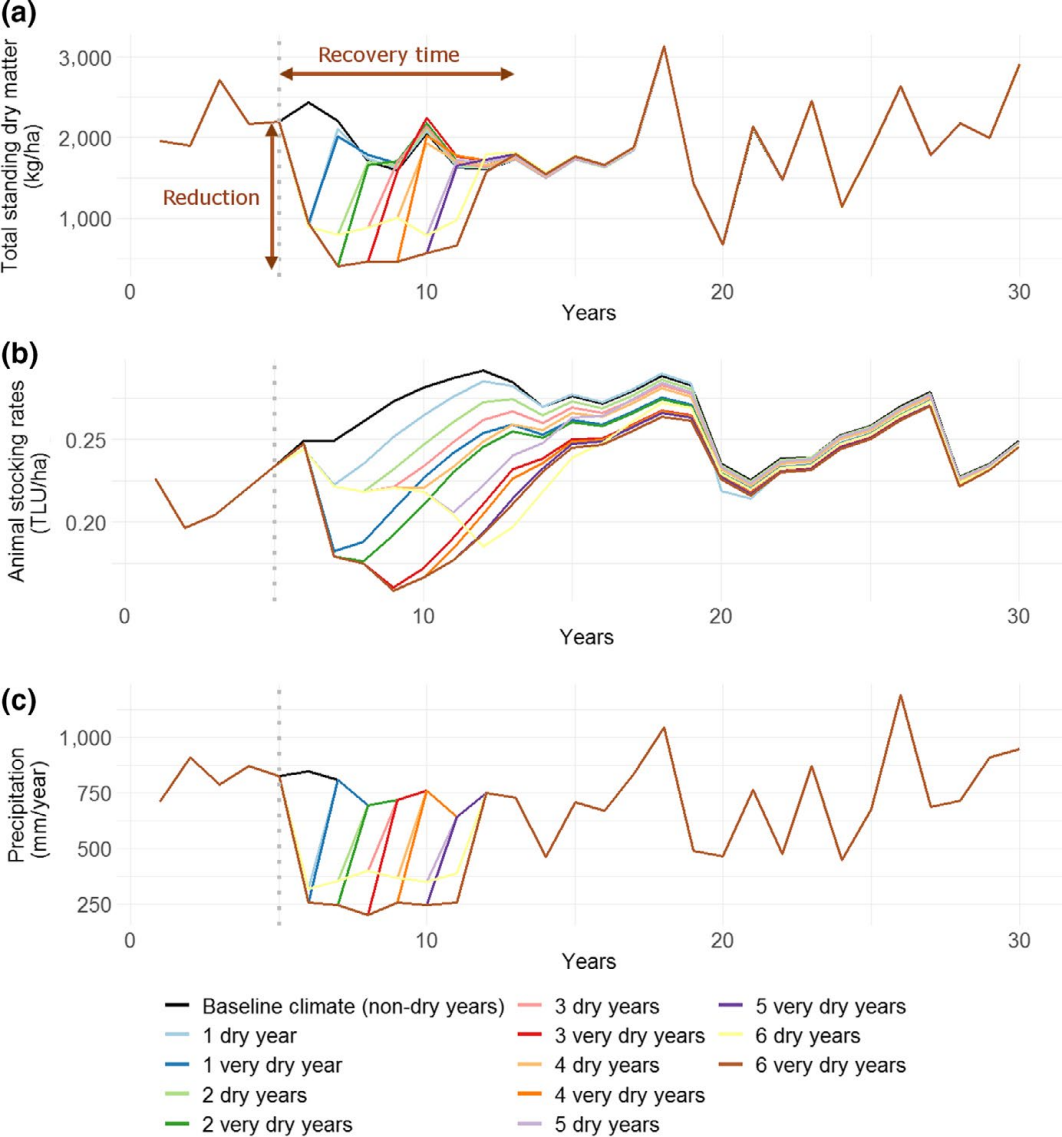
Mean annual total standing dry matter (a), animal stocking rates (b) and corresponding mean annual precipitation (c) under different imposed drought intensities and durations (Set 1 of climate scenarios). Farming practices considered: baseline practices. ‘1 dry year’: one dry year imposed at year 6; ‘2 dry years’: two consecutive dry years imposed at years 6 and 7, etc. See Appendix S2 for the climate scenarios mean annual precipitation, CVP‐intra and CVP‐inter. CVP‐inter, interannual coefficient of variation of precipitation; CVP‐intra, intra‐annual coefficient of variation of precipitation; TLU, tropical livestock units

These generated years were then classified depending on their precipitation level. ‘Very dry’ years correspond to years statistically occurring in the data set once every 100 years (precipitation below 281 mm/year), ‘dry’ years to years occurring once every 10 years (281–432 mm/year) and ‘non‐dry’ years to years that were neither ‘very dry’ nor ‘dry’ (>432 mm/year). We then selected years from this data set to generate 13 time series of 30 years. The baseline scenario included non‐dry years only and was only used to estimate herd recovery times after drought events (see section ‘Proxies to characterize the herd dynamics2.7’). The other scenarios included varying numbers of consecutive dry and very dry years (‘drought period’) which were imposed from year 6. The non‐dry years in these other scenarios were the same as in the baseline. Figure 4 shows the list of these 13 scenarios. The 432 mm/year precipitation threshold that differentiated ‘non‐dry’ and ‘dry’ years was relatively consistent with the years considered in the region as drought years for livestock production. Indeed, the ‘Queensland 1990s drought’ (1992–1996) was associated with precipitations below 437 mm/year in the Wambiana region (Stehlik, Gray, & Lawrence, 1999). The drought period 2001–2006 showed precipitations below 490 mm/year (O'Reagain & Bushell, 2011) and the drought period 2013–2015 showed precipitations below 487 mm/year (State of Queensland, 2015). Other studies on Australian grazing systems identified the first year of extended drought periods when annual precipitation was >70% of the mean (here 70% × 653 = 457 mm/year, Table 2; McKeon, Hall, Henry, & Watson, 2004; Stafford Smith et al., 2007). Given the likelihood of droughts becoming more severe under climate change (Watterson et al., 2015), we chose a period of up to six consecutive ‘dry’ and ‘very dry’ years to explore herd recovery times.

#### 2.5.2 Set 2—Precipitation mean and variability effects on herd dynamics

As a complementary approach, we also generated a second set of scenarios to capture precipitation long‐term trends. We used MarkSim to generate 15 scenarios that covered a range of precipitation means (373–1,157 mm/year) as well as inter‐ and intra‐annual precipitation patterns (0.27–0.44 and 0.24–1.38, respectively) to represent possible effects of climate change. The 15 time series were 30 years long, 30 years being the standard reference period to define a climate (WMO, 2018). The range of scenarios tested (Table 3 and Appendix Figures S2–S7) aimed to cover the ‘uncertainty space’ as to how precipitation patterns may change in the future (Sillmann, Kharin, Zwiers, Zhang, & Bronaugh, 2013; Warszawski et al., 2014). For northern Australia, future precipitation changes are uncertain with some models showing a wetting trend although overall a drying trend is favoured (Watterson et al., 2015). Trends in variability are also uncertain, though there is a high level of confidence that heavy rainfall events will be more intense.

### 2.6 Proxies to characterize the climate scenarios

The three variables used to characterize the climate scenarios were mean precipitation (mm/year), and inter‐ and intra‐annual coefficient of variation of precipitation (CVP‐inter and CVP‐intra, respectively). CVP‐inter was calculated as the standard deviation of the annual precipitation divided by the mean annual precipitation for the full time series. CVP‐intra for the time series was calculated as the standard deviation of the average precipitation for the 12 months of the year divided by the mean of these 12 monthly averages.

### 2.7 Proxies to characterize the herd dynamics

The main outputs described in this modelling study were time‐series mean forage TSDM (kg/ha) and herd stocking rates (TLU/ha). We also considered time‐series mean animal liveweight (kg), mortality (TLU ha^−1^ year^−1^), mortality rates (%), sales (TLU ha^−1^ year^−1^), sales rates (%), total enteric methane emissions (kg CO_2_‐eq ha^−1^ year^−1^) and enteric methane emissions intensities (kg CO_2_‐eq/kg liveweight sold). We also assessed TSDM and herd stocking rate reductions and recovery times under the first set of climate scenarios (Set 1). These two variables provide information on the rangeland system ability to absorb and recover from the effect of droughts, which constitutes one of the components of the system's resilience concept (IPCC, 2012). In this study, the reduction in TSDM and stocking rates was defined as the percentage of drop from the year 5 variable's value to its lowest value. A recovery time was defined as the number of years it takes for a variable's value of a specific time series to reach the baseline value (stocking rate values rounded at two digits after the decimal point). Figure 4 shows a graphical representation of the reduction and recovery time variables.

### 2.8 Statistical analyses

Given the complexities of the forage and herd system dynamics model (i.e. involving non‐linear relationships as well as feedbacks), we undertook statistical analyses to describe some of the grazing system's response to climate and farming scenarios. Regression tables are provided in Appendix S3.

We used linear regressions to model the relationship between explained variables (e.g. mean TSDM for the time series) and explanatory variables related to climate and farming scenarios (e.g. mean precipitation, CVP‐inter, CVP‐intra, forage production type for the time series). The statistical model was:

(1) $$E\left(Y|X_{1},\ldots ,X_{p}\right)=\alpha +\beta_{1}X_{1}+\cdots +\beta_{p}X_{p}+\varepsilon$$

where *Y* is the response measurement, *X*_*i*_ is the explanatory variable *i*, *α* is the intercept, *β*_*i*_ is the slope or coefficient and *ε* the errors.

To summarize the contribution of the explanatory variables alone to the explained variable variance, we calculated the coefficient of determination (*R* ^2^) of the explanatory variable *X*_*i*_ for the model including only the explanatory variable *i*:

(2) $$E\left(Y\left|X_{i}\right.\right)=\alpha +\beta_{i}\times X_{i}+\varepsilon$$

where *Y* is the response measurement, *X*_*i*_ is the explanatory variable *i*, *α* is the intercept, *β*_*i*_ is the slope or coefficient and *ε* the errors.

## 3 RESULTS

### 3.1 Effect of drought period on herd stocking rate reduction and recovery time

The effect of droughts on herd dynamics was studied by imposing different drought intensities and durations (Set 1 of climate scenarios—Figure 4). The farming practices considered in this section were baseline practices (TSDM = 2,000 kg/ha, no feed supplementation).

TSDM and animal stocking rates were significantly affected by drought events. We found that the larger the intensity of the drought, the larger the reduction in stocking rates. Similarly, the longer the drought, the longer the recovery time, with herd recovery times longer than a couple of decades in some cases (see section ‘Proxies to characterize the herd dynamics2.7’ for stocking rate reduction and recovery time definitions).

Stocking rate recovery times were not only affected by drought durations but also by drought intensities. For instance, after one dry year and one very dry year, stocking rates took 8 and 18 years, respectively, to reach the baseline values in the absence of proactive restocking through animal purchases. In contrast, TSDM recovery times were almost only responsive to drought durations and not intensities. It could be explained by that fact that the model does not represent long‐term feedbacks of unsustainable forage utilization rates on forage productive capacity. This is further detailed in the Discussion4 section. The interaction effect between drought intensity and duration was statistically negligible (Appendix S3).

Reductions in TSDM were proportionally larger than reductions in stocking rates. For instance, TSDM dropped by 74% and stocking rates by 29% when six consecutive very dry years were imposed. However, the herd took up to three times longer to recover than pasture. For instance, recovery times were up to 24 years for stocking rates as compared to 7 years for TSDM.

Stocking rates were influenced by herd sales and mortality. While mean sales rates under drought periods were not very different from the baseline, interannual variations in sales rates increased to a greater extent, highlighting the increased complexity of farmers selling routines during droughts. For instance, the coefficient of variation of sales rate was 0.37 under a six consecutive very dry years period (mean sales rate for that climate scenario: 23%), much more than the 0.06 estimated for the 6 year baseline (mean: 22%), and this due to most sales occurring in the first couple of years after which there were not many animals left to sell. Mortality rates and interannual variations in mortality rates also increased under drought periods. The coefficient of variation of mortality rate was 0.67 over a six consecutive very dry years period (mean mortality rate: 6%), much more than the 0.02 estimated for the 6 year baseline (mean: 1%).

### 3.2 Effect of precipitation mean and variability on herd dynamics

**Figure 5:**
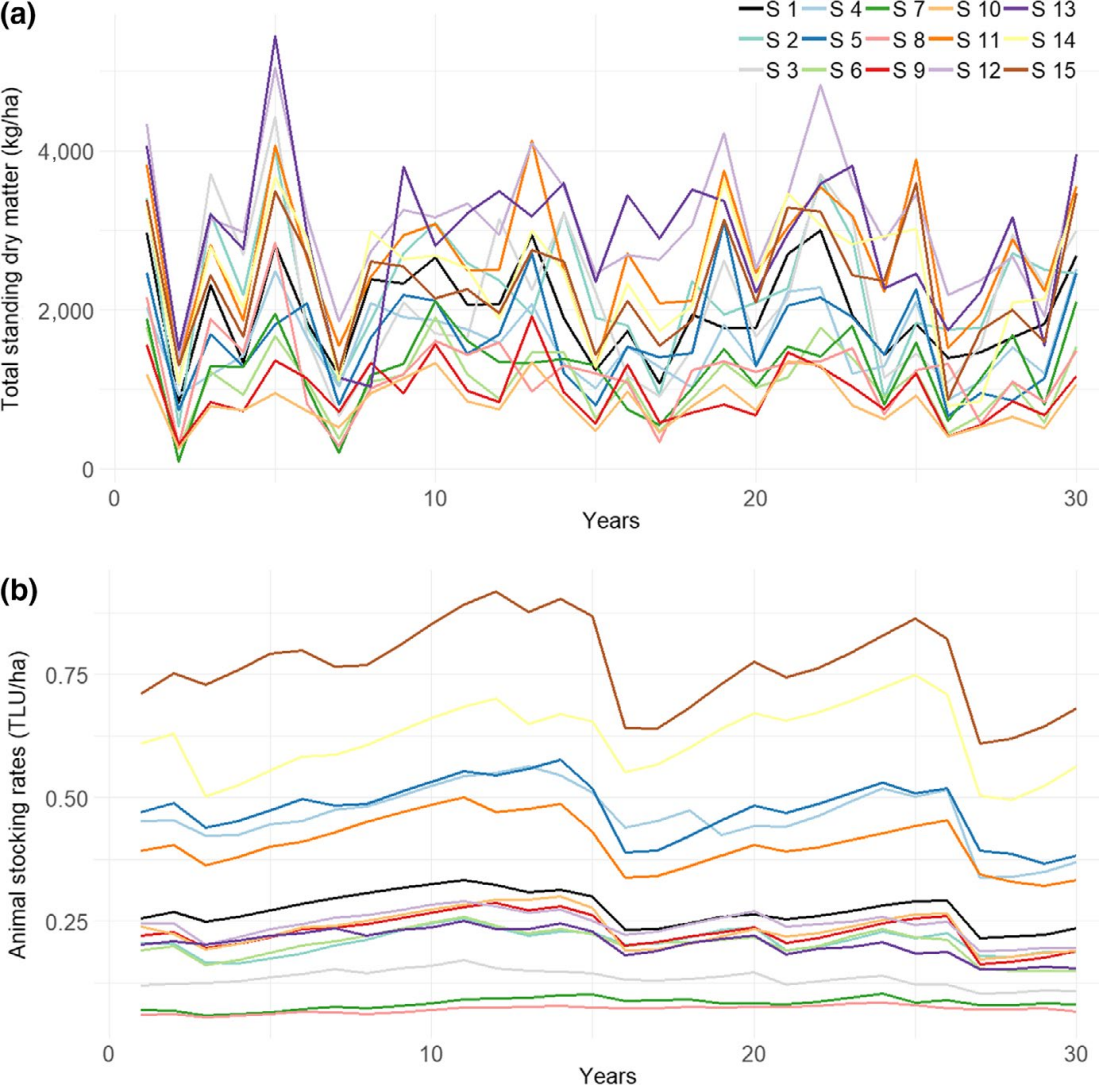
Mean annual total standing dry matter and animal stocking rates under 15 climate scenarios (Set 2 of climate scenarios). Farming practices considered: baseline practices. See Table 3 and Appendix S2 for the climate scenarios mean annual precipitation, CVP‐intra and CVP‐inter. CVP‐inter, interannual coefficient of variation of precipitation; CVP‐intra, intra‐annual coefficient of variation of precipitation; TLU, tropical livestock units

In this section, we study forage and herd dynamics under a range of precipitation means and variabilities (Set 2 of climate scenarios, Figure 5). The farming management practices considered were baseline practices (TSDM = 2,000 kg/ha, no feed supplementation).

The 30 year long‐term time series average for TSDM, stocking rates, sales and mortality were significantly correlated with the time‐series mean precipitation and CVP‐intra (*R* ^2^ > 0.90, *p* < 0.05). High *R* ^2^ indicated very small effects of the interactions between climate variables on the explained variables. Based on individual linear regressions, we found that a decrease of 20% in the time‐series mean precipitation was associated with a decrease of 19% of mean TSDM (*R* ^2^ = 0.92, *p* < 0.05). A decrease of 20% of the time‐series mean precipitation or an increase of 20% CVP‐intra was associated with a decrease of 18% and 19% of mean stocking rates (*R* ^2^ = 0.30–0.51, *p* < 0.05). As for sales rates, they were significantly negatively related to CVP‐intra and CVP‐inter, and positively related to mean precipitation (*R* ^2^ = 0.94, *p* < 0.05). Animal liveweight was negatively related to CVP‐inter (*R* ^2^ = 0.78, *p* < 0.05) and relationships for mortality rates were inconclusive (*R* ^2^ = 0.13).

**Figure 6:**
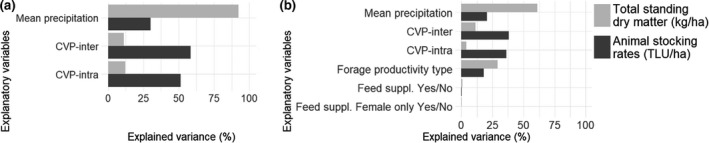
Contribution of climate and management variables to the variance in time‐series mean total standing dry matter (light grey) and animal stocking rates (dark grey). In (a), only baseline farming practices were considered. In (b), both climate and management variables were considered. CVP‐inter, interannual coefficient of variation of precipitation; CVP‐intra, intra‐annual coefficient of variation of precipitation

To gain further insights as to the contribution of the different climate variables to forage and herd dynamics, we assessed their contribution to the variance of TSDM and stocking rates (Figure 6a). Most of the variance in mean TSDM among the 15 climate scenarios was explained by mean precipitation of the time series (92%), followed by CVP‐intra (12%) and CVP‐inter (11%), when these explanatory variables were considered without their interaction with other variables. Most of the variance in mean stocking rates was explained by CVP‐inter (58%), followed by CVP‐intra (51%) and mean precipitation (30%).

The positive effects of higher mean precipitation on stocking rates could be reduced by increased climate variability and vice versa (Table 3). For example, for time series with mean precipitation over 1,067 mm/year (75th percentile—in that case, CVP‐inter was 0.28–0.35), CVP‐intra of 0.27–0.43 was associated with a mean stocking rate of 0.69 TLU/ha while CVP‐intra of 1.22–1.38 was associated with a mean stocking rate of 0.22 TLU/ha.

**Table 3:** Mean total standing dry matter (A) and animal stocking rates (B) under 15 climate scenarios (Set 2 of climate scenarios)

Total standing dry matter (kg/ha)	Animal stocking rates (TLU/ha)	Mean precipitation (mm/year)	CVP‐inter	CVP‐intra	Climate scenario
841	0.23	373	0.35	0.24	S 10
982	0.22	415	0.34	0.5	S 9
1,093	0.2	434	0.36	0.75	S 6
1,225	0.07	410	0.42	1.31	S 8
1,254	0.09	433	0.39	1.21	S 7
1,585	0.45	712	0.27	0.51	S 4
1,624	0.49	742	0.33	0.32	S 5
1,983	0.26	751	0.29	0.86	S 1
2,177	0.14	780	0.44	1.36	S 3
2,261	0.21	839	0.35	1.21	S 2
2,341	0.74	1,091	0.28	0.27	S 15
2,411	0.64	1,067	0.29	0.43	S 14
2,691	0.4	1,054	0.28	0.81	S 11
2,923	0.21	1,087	0.35	1.38	S 13
3,133	0.24	1,157	0.31	1.22	S 12

Note

Farming practices considered: baseline practices. The darker the shade of grey, the higher the variable value.

Abbreviations: CVP‐inter, interannual coefficient of variation of precipitation; CVP‐intra, intra‐annual coefficient of variation of precipitation.

Interannual variability in TSDM was significantly related to CVP‐inter and CVP‐intra (*R* ^2^ = 0.87, *p* < 0.05) while interannual variability in SR was not significantly related to any variable (*R* ^2^ = 0.65). Interannual variability in TSDM (from 0.27 to 0.45, mean: 0.36) was on average 6% higher than CVP‐inter (from 0.27 to 0.44, mean: 0.34) for each of the time series, which is similar to findings from Le Houérou et al. (1988) for case studies with comparable CVP‐inter. Interannual variability in stocking rate (from 0.07 to 0.13, mean: 0.10) was on average 71% lower than interannual variability in TSDM and 72% lower than CVP‐inter.

### 3.3 Effect of intensification strategies on herd dynamics

In this section, we show the effects of forage and feed supplementation strategies on herd dynamics, taking into account model outputs averaged over the second set of climate scenarios (Set 2—same as in the section above). We also discuss how farming practices compare to climate variables in terms of their impact on forage and herd characteristics.

The combined intensification strategies (high forage productivity and feed supplementation) gave the greatest response in annual stocking rate, sales and sales rates, closely followed by improved forages (Table 4). For instance, under the combined intensification strategy, the stocking rate was 0.62 TLU/ha as compared to 0.31 TLU/ha under baseline management. Also, animal numbers sold were 0.18 TLU ha^−1^ year^−1^ as compared to 0.09 TLU ha^−1 ^year^−1^, and the sales rate was 31% as compared to 28%. Feed supplementation of the whole herd increased animal liveweight gain (from 130 to 143 kg TLU^−1^ year^−1^) and stocking rates (from 0.31 to 0.32 TLU/ha) while reducing interannual variation in animal stocking rates (from 0.10 to 0.03) and mortality rates (from 3.7% to 3.1%). The feeding strategy also resulted in lower mean TSDM (1,695 kg/ha) and higher interannual variation in TSDM (0.64) as compared to the baseline management (1,902 kg/ha, 0.36). This was driven by a higher total herd forage intake from reduced mortality rates, reduced declines in fertility rates and reduced declines in intake per animal, particularly during dry years.

**Table 4:** Mean forage and herd characteristics under different intensification scenarios

	Baseline management	Forage productivity ×1.5	Forage productivity ×2	Feed suppl. female only	Feed suppl. female + male	Combined forage productivity ×2 and feed suppl. female + male
Annual total standing dry matter (kg/ha)	1,902 *(733)*	2,855 *(1,100)*	3,808 *(1,467)*	1,798 *(782)*	1,695 *(822)*	3,402 *(1,645)*
Annual animal stocking rates (TLU/ha)	0.31 *(0.20)*	0.45 *(0.29)*	0.60 *(0.39)*	0.33 *(0.16)*	0.32 *(0.15)*	0.62 *(0.30)*
Interannual variation in total standing dry matter	0.36 *(0.05)*	0.36 *(0.05)*	0.36 *(0.05)*	0.45 *(0.18)*	0.64 *(0.46)*	0.64 *(0.45)*
Interannual variation in animal stocking rates	0.10 *(0.02)*	0.10 *(0.02)*	0.10 *(0.02)*	0.04 *(0.01)*	0.03 *(0.01)*	0.03 *(0.01)*
Animal mortality (TLU ha^−1^ year^−1^)	0.01 *(0.01)*	0.02 *(0.01)*	0.02 *(0.01)*	0.01 *(0.00)*	0.01 *(0.00)*	0.02 *(0.01)*
Annual animal mortality rate (%)	3.7 *(0.00)*	3.9 *(0.00)*	3.9 *(0.00)*	3.1 *(0.00)*	3.1 *(0.00)*	3.2 *(0.00)*
Animal liveweight gain (kg TLU^−1^ year^−1^)	130 *(4.75)*	131 *(4.78)*	131 *(4.84)*	135 *(7.39)*	143 *(5.05)*	143 *(5.07)*
Animal sales (TLU ha^−1^ year^−1^)	0.09 *(0.06)*	0.13 *(0.09)*	0.17 *(0.12)*	0.09 *(0.05)*	0.09 *(0.05)*	0.18 *(0.10)*
Annual animal sales rate (%)	28 *(0.04)*	29 *(0.04)*	30 *(0.03)*	30 *(0.02)*	30 *(0.02)*	31 *(0.02)*
Methane (kg CO_2_‐eq ha^−1^ year^−1^)	286 *(191)*	428 *(287)*	570 *(383)*	321 *(170)*	323 *(160)*	640 *(321)*
Methane intensity (kg CO_2_‐eq/kg liveweight sold)	14.1 *(1.59)*	13.8 *(1.26)*	13.6 *(1.11)*	13.8 *(0.52)*	14.5 *(0.65)*	14.1 *(0.56)*

Note

Averages estimated over all 15 climate scenarios (Set 2 of climate scenarios, mean precipitation = 756 mm/year, CVP‐inter = 0.34, CVP‐intra = 0.83). Standard deviations for the set of climate scenarios are between parentheses.

Abbreviation: CVP‐inter, interannual coefficient of variation of precipitation; CVP‐intra, intra‐annual coefficient of variation of precipitation; TLU, tropical livestock units.

Linear regressions including both climate and management variables showed that mean TSDM, sales rates, mortality rates and mortality during the time series were significantly related to mean precipitation, CVP‐inter, CVP‐intra, forage productivity type and feed supplementation (*R* ^2^ > 0.81, *p* < 0.05). Stocking rates and sales were significantly related to all variables, except feed supplementation strategies (*R* ^2^ > 0.81, *p* < 0.05). Liveweight and interannual variability in TSDM were significantly related to all variables except CVP‐intra and forage productivity type (*R* ^2^ > 0.40, *p* < 0.05). Interannual variability in stocking rate was significantly related to mean precipitation and feed supplementation strategies (*R* ^2^ = 0.84, *p* < 0.05). The differences in TSDM and SR between feed supplementing both males and females as compared to females only were inconclusive.

We also assessed how climate variables compared to farming scenarios in explaining the variance of TSDM and stocking rates (Figure 6b). Most of the variance in TSDM among the 15 climate scenarios was explained by mean precipitation (61%), followed by forage productivity type (29%), CVP‐inter (11%) and CVP‐intra (4%), when these variables were assessed independent of interactions with other variables. Feed supplementation contributed to less than 1% of the variance. Most of the variance in mean stocking rates was explained by climate variables (CVP‐inter: 38%, CVP‐intra: 36%, mean precipitation: 21%), followed by forage productivity type (18%) and feed supplementation (<1%).

The positive effects of intensification strategies on stocking rates could be reduced by decreased mean precipitation or increased climate variability and vice versa. For example, time series with high forage productivity (×2), mean precipitation of 1,054–1,091 mm/year and CVP‐inter of 0.28 resulted in a mean stocking rate of 1.46 TLU/ha under CVP‐intra = 0.27 and 0.79 TLU/ha under CVP‐intra = 0.81.

### 3.4 Effect of stocking rate levels on herd size reductions and enteric methane emissions

**Figure 7:**
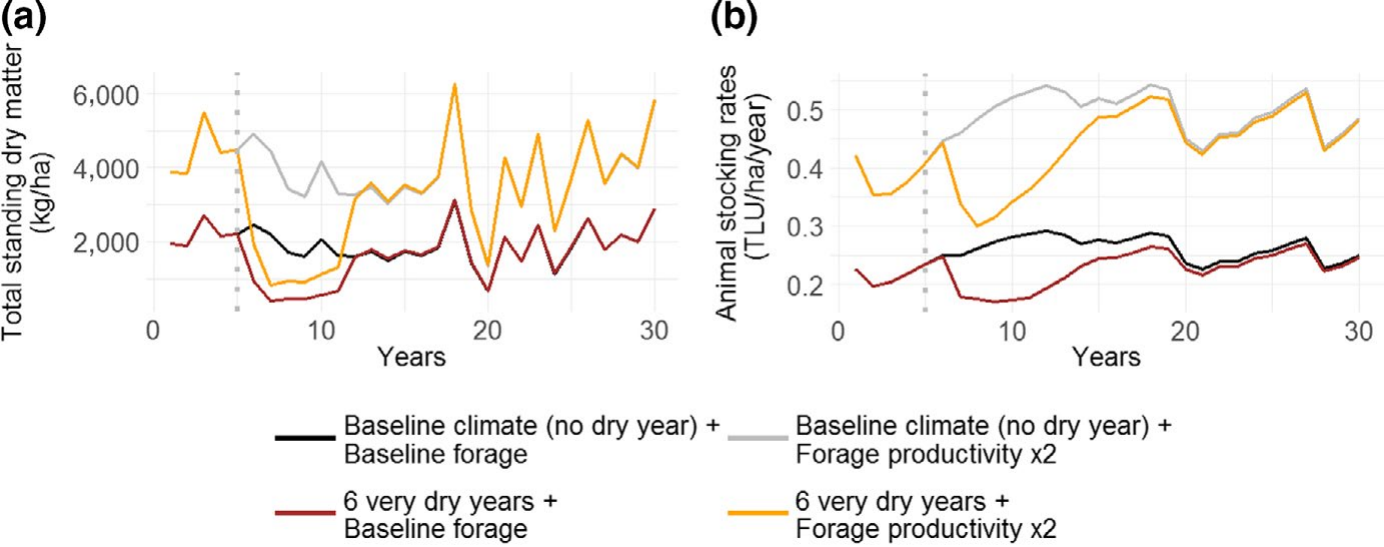
Mean annual total standing dry matter (a) and animal stocking rates (b) under different climate and forage productivity scenarios. No feed supplementation was provided to the herd. See Appendix S2 for more details about Set 1 of climate scenarios. TLU, tropical livestock units

Graphical interpretations showed that higher stocking rates from intensification strategies and favourable climates resulted in larger reduction in animal numbers in absolute terms (see section ‘Proxies to characterize the herd dynamics2.7’ for stocking rate reduction definition). For instance, high forage productivity types resulted in a larger reduction in annual stocking rates under six consecutive very dry years when compared to the baseline forage productivity type (−0.11 TLU/ha and −0.06 TLU/ha, respectively, Set 1 of climate scenarios, Figure 7). However, in relative terms, both forage types resulted in a decrease of 27% of the herd size. Similarly, stocking rates under a high mean precipitation scenario dropped by 0.23 TLU/ha between years 15 and 16 (from 0.87 to 0.64 TLU/ha, Set 2 of climate scenarios—S 15: mean = 1,091 mm/year, precipitation dropped from 1,127 to 575 mm/year between years 14 and 15, Figure 5). The reduction was lower under baseline climate scenario (from 0.30 to 0.23 TLU/ha, S 1: mean = 751 mm/year, precipitation dropped from 625 to 398 mm/year between years 14 and 15).

Total production of enteric methane increased under intensification scenarios and favourable climates due to a higher number of animals sustained on the land. For instance, the combined intensification strategies, which resulted in higher productivities, also led to methane emissions of 640 kg CO_2_‐eq ha^−1^ year^−1^ as compared to 286 kg CO_2_‐eq ha^−1^ year^−1^ under baseline management (Set 2 of climate scenarios, Table 4). In contrast, the intensity of methane production (i.e. the amount of methane per kilogram of beef produced) decreased under improved forage scenarios, but not enough to offset the overall methane emissions from higher stocking rates. This is because the intensification strategies mostly affected carrying capacity rather than production per head. Feed supplementation of the whole herd resulted in increased methane intensities as it stimulated forage intake during the dry season. Similarly, climate scenarios with mean precipitation over 1,067 mm/year (75th percentile) resulted in 371 kg CO_2_‐eq ha^−1^ year^−1^ while climate scenarios with mean precipitation lower than 434 mm/year (25th percentile) resulted in mean methane emissions of 160 kg CO_2_‐eq ha^−1^ year^−1^ under baseline management. Methane production intensity was lower in the second case (>1,067 mm/year: 13.8 kg CO_2_‐eq/kg liveweight sold; <434 mm/year: 14.7 kg CO_2_‐eq/kg liveweight sold).

## 4 DISCUSSION

In this semi‐arid Australian rangeland case study, forage production and animal stocking rates were significantly impacted by drought events and long‐term climate trends. Increases in precipitation means were favourable to grazing systems productivity while increases in climate variability negatively affected herd sizes. Although forage was proportionally more responsive to climate variability than the herd size, herds recovered more slowly after drought events, taking up to decades in some cases, in the absence of stock purchases to accelerate herd recovery. Farming intensification strategies increased long‐term herd sizes but had less of an impact than climate on the variance in animal stocking rates. Similar to favourable climates, intensification also resulted in larger reductions in animal numbers during droughts and raised total enteric methane emissions.

Although the herd‐forage model was developed to allow the testing of different potential scenarios, the current version of the model was not aimed at capturing the operational diversity and complexities of actual livestock enterprises in their entirety. In common with any model of a complex system, it was developed with a specific purpose and is underlined by a number of simplifying assumptions (see below and Appendix S1). These simplifications could also in part explain the relatively high goodness‐of‐fit (*R* ^2^) of some of the regressions presented in this paper as compared to those that can be estimated from empirical grazing studies (e.g. *R* ^2^ up to 0.36 for linear relationships between annual TSDM and precipitation found in the Wambiana region; O'Reagain & Bushell, 2011).

We found that forage and herd characteristics were significantly affected by drought intensities and durations. Series of consecutive dry years are not unusual in northern Queensland as exemplified by the periods 1992–1996, 2001–2004 and 2013–2015, which showed annual precipitations below 500 mm (see annual precipitation patterns since 1900 in Appendix S2, Bureau of Meteorology, 2018). The duration and frequency of these dry years, which are usually associated with El Niňo events, may also increase in the near future, although large climate uncertainties remain justifying the sensitivity approach undertaken in this study (Sillmann et al., 2013; Watterson et al., 2015, see example of projected climate uncertainties in Appendix S2). We found that with six consecutive years of very intense dryness, even with early decision‐making in response to declining forage availability, the herd stocking rate decrease by 29%, and it took 24 years to fully recover herd numbers in the absence of additional animal purchases. In that case, the herd market liveweight value dropped by 31% (from 147AU$ to 101AU$ per hectare; prices from Meat & Livestock Australia, 2017). In this modelling study, the herd was managed to ensure pasture utilization rates that are considered for the region as economically and environmentally sustainable (Ash et al., 2011; Hunt, 2008; O'Reagain & Bushell, 2011; O'Reagain et al., 2014). While this is a strategy practised by a number of farmers in the region, other social and economic drivers can influence stocking decisions (Marshall, 2015). Some pastoralists may be inclined to keep their livestock for longer than ecologically desirable because they have invested considerable time and energy in building a herd with desired attributes. This is especially so if prices are low. Ultimately, this strategy can result in sudden emergency sales if dry conditions persist. Pastoralists who conservatively manage their pasture resource with longer term low utilization rates typically need to destock less than those producers who have higher overall utilization rates and are more prone to forage deficits when there is a succession of dry seasons. For example, in an experiment on grazing management in northern Queensland, during an extended drought that started in 2000, the moderate stocking rate treatment (~0.22 TLU/ha) could be sustained without any stock reductions, while the high stocking rate treatment showed a cattle herd number reduction by 47% between 2004 and 2006 (from 0.45 to 0.24 TLU/ha) and the variable stocking treatment that proactively matched stock numbers to forage supply had to reduce cattle numbers by 62% between 2000 and 2004 (0.45–0.17 TLU/ha, O'Reagain & Bushell, 2011). Emergency sales can exacerbate livestock market price drops (Meat & Livestock Australia, 2015) as well as land degradation and animal welfare issues. For instance, pastoralists may not be able to sell their animals because of abattoirs reaching full capacity (Commonwealth of Australia, 2016b).

In this study, the herd was managed to prevent excessive land degradation. Herd size reductions and recovery times are likely to be even longer under long‐term overstocking and drought events due to negative feedback effects of forage utilization rates and droughts on forage productive capacity (represented by forage structures and pasture species composition, not modelled here). In some parts of Western Australia, particularly in the Gascoyne–Murchison region, extreme overgrazing over the years has resulted in the land not being productive enough for livestock enterprises (CSIRO, 1978; Wilcox & McKinnon, 1974). Much of this land has been converted to either conservation use or highly modified livestock enterprises that rely on other forms of income such as tourism.

In terms of long‐term climatic trends, decreases in precipitation means or increases in interannual and intra‐annual precipitation variability negatively affected herd sizes in this Australian case study. These trends are captured at the global scale. For example, the less climatically stable areas have the lowest cattle densities (Sloat et al., 2018). Historical climate records show that CVP‐inter in Wambiana increased by 34% between 1915 and 2003 and mean precipitation by 5% (mean of 1900–1930 and 1989–2018). As for future climatic trends, these are highly uncertain (IPCC, 2000; Sillmann et al., 2013; Warszawski et al., 2014), as highlighted by projections from General Circulation Models for the Wambiana region (Warszawski et al., 2014; Watterson et al., 2015). For example, mean precipitation in 2085 is projected to increase by 34% as compared to 2000 according to the climate model NorESM1‐M under the Representative Concentration Pathway (RCP) 8.5 and decrease by 25% according to HadGEM2‐ES under RCP 8.5 (means of 1986–2015 and 2070–2099; van Vuuren et al., 2011; Warszawski et al., 2014). Similarly, CVP‐inter is projected to increase by 20% according to HadGEM2‐ES under RCP 2.6 and by 8% according to HadGEM2‐ES under RCP 8.5. Not modelled in this study, climate change‐driven variations in temperature, solar radiation and atmospheric carbon dioxide concentrations, as well as vegetation species composition, land competition, water, heat stress, fires and diseases may also impact livestock production and rangeland ecosystems dynamics. Notably, complex ecosystems transitions between states of equilibrium (livestock populations driven primarily by density‐dependent interactions via competition for food resources) and non‐equilibrium (livestock populations driven primarily by abiotic factors, such as precipitation), while not captured in this model, may occur under changing climates (Ellis & Swift, 1988). Other drivers of change in livestock systems and in broader development trends also add to future uncertainties and need to be further studied.

While this study focusses on a northern Australian beef system, other rangelands systems are also constrained by low precipitation and high and increasing climate variability (e.g. Namibia, north–east Kenya and south Argentina; Sloat et al., 2018). These regions are mainly located in developing countries where livestock is crucial for food access or the economy (Sloat et al., 2018).

Intensification strategies such as feed supplementation and improved pastures can be key strategies to adapt to climate change as they may result in farm production gains, greater herd size being carried with more animals being turned off for sale. While the current implementation of such strategies varies globally, examples are found around the world (FAO, 2007; Rao et al., 2015). These changes in farming practices contribute, in different ways, to herd dynamics as compared to climate change. For instance, in this study, climate had the largest contribution to the stocking rates variance, followed by forage. Feeding practices had a negligible contribution. As highlighted in this study, intensification strategies may also increase the sensitivity of the herd to drought events as well as total enteric methane emissions and come at other costs that might not be offset by increased production levels. Detailed analyses of the economic, labour and environmental trade‐offs of such interventions and enabling environments (markets, policies, social and human capital) need to be assessed within a context of increased climate pressures, complex financial market fluctuations and social environments (Godde, Garnett, Thornton, Ash, & Herrero, 2018; Stafford Smith et al., 2007). Low forage nutritional quality in extensive rangeland systems tend to result in high methane emission intensities as compared to other livestock production systems (Ash et al., 2015; Charmley, Stephens, & Kennedy, 2008; Herrero et al., 2013). Intensifying the production systems generally increases total methane emissions and can reduce emissions intensity (Ash et al., 2015; White, Snow, & King, 2010). Management decisions can be made along the intensification spectrum to balance productivity and profitability objectives versus environmental ones. For instance, Ash et al. (2015) found that modelled intensification practices such as protein supplementation or legume sowing improved pastures, genetics or rumen functions could increase farm enterprise profitability under historical climate in northern Queensland, while decreasing methane emission intensities. They also found that if some of the gains in profit from introducing technologies were foregone by reducing the herd size so that methane production per hectare does not increase over the baseline, then maximum net profit was reduced by about 10% but it was still considerably higher than the baseline management strategy. The profitability of intensification strategies can, however, vary among global rangelands. For instance, Hatch and Stafford Smith (1997) found that feed supplementation as a drought management strategy was not economically viable in a semi‐arid South African rangeland modelling case study. Farmers may be required to adjust their practices and stocking rate targets on a more frequent basis to maximize both short‐ and long‐term economic benefits while preventing land degradation. In Australia, farmers have been maintaining low stock numbers and pasture utilization rates to limit the effects of high climate variability (Landsberg, Ash, Shepherd, & McKeon, 1998). Moving livestock to take advantage of spatial heterogeneity in forage availability has also been a key adaptation strategy under high climate variability in many rangelands (e.g. southern Africa, Mongolia, China). However, this option is increasingly challenged as landscapes become fragmented and communal land tenure systems shift to semi‐commercial ones (Dalintai, Gauwau, Yanbo, Enkhee, & Shurun, 2012; Dube & Pickup, 2001; Galvin, Reid, Behnke, & Hobbs, 2008; Hobbs et al., 2008; Hruska et al., 2017). In Australian rangelands, the use of ‘agistment’ where cattle are moved from a drought area to other privately run properties that have adequate pasture and graze for a fee is common practice. However, the shortage of productive land during widespread droughts often limits the possibility and economic viability of such a mobility strategy. Climate forecasting and drought monitoring initiatives are emerging (e.g. national monitoring in Australia, National Integrated Drought Information System and Drought Portal in the United States, Famine Early Warning Systems Network) and may increasingly assist farmers in their stocking management under climate change. Further research as to the economic and environmental value of such information as well as the role of farmers insurances need to be assessed. Investments in the adaptive capacity of farmers and rural communities will also be necessary (Crimp et al., 2010; Marshall, Stokes, Webb, Marshall, & Lankester, 2014). For instance, information and communications technologies are having an increasing influence on formerly remote and isolated communities and farmers' capability to absorb information, analyse it and apply it is often a constraint. While this was not the focus of this study, further detailed research into the value of changes in management strategies over time in response to changing conditions is much needed. In addition to feed supplementation, improving pastures and altering stocking, other management options such as changing livestock genetics or breeds, adjusting fire management practices, increasing shade or water points exist.

By exploring the extent of potential climate impacts on grazing systems productivity, adaptation options as well as implications for enteric methane emissions, this study addresses the three pillars of climate‐smart agriculture necessary to achieve food security and other United Nations Sustainable Development Goals (i.e. productivity, adaptation and mitigation pillars; Lipper et al., 2014). The system dynamics model presented provides the basis for a flexible herd‐forage structure to which can be added other effects of climate variables as well as management options of varying complexity. As such, it offers potential for subsequent research that may cover different agroecosystems and managements. Global grasslands are important providers of ecosystem services and a major source of food and income in most parts of the world. In the face of global warming and the sensitivity of grazing systems to climate, the existing suite of adaptation strategies and coping range that have been developed solely in response to existing variability may not be enough (Ash, Thornton, Stokes, & Togtohyn, 2012). Context‐specific and timely technical options and policy and enabling environment are urgently needed to facilitate the widespread adaptation required to cope with climate change. Deepening our understanding of climate change impacts on grazing systems and pathways for adaptation and mitigation is a necessary step in this process.
