# Supplementary material for: Climate change and variability impacts on grazing herds: Insights from a system dynamics approach for semi‐arid Australian rangelands
Source: Glob Chang Biol. 2019 Jun 24;25(9):3091–109. doi: 10.1111/gcb.14669 (PMC6771763; doi:10.1111/gcb.14669)
Supplement: Supplementary file 1 [file GCB-25-3091-s001.docx]

Supplementary information - Climate change and variability impacts on grazing herds: Insights from a system dynamics approach for semi-arid Australian rangelands

Godde, C.M.^1, 2^, Dizyee, K. ^1^, Ash, A.^1^, Thornton, P.^3^, Sloat, L.^4^, Roura, E^2^, Henderson, B.^5^, Herrero, M.^1^

^1^ Commonwealth Scientific and Industrial Research Organisation, St Lucia, QLD 4072, Australia

^2^ The University of Queensland, St Lucia, QLD 4072, Australia

^3^ CCAFS, International Livestock Research Institute (ILRI), PO Box 30709, Nairobi 00100, Kenya

^4^ University of California Irvine, 92697 Irvine, USA

^5^ Organisation for Economic Co-operation and Development, 75775 Paris, France

Appendix S1 - Forage and herd model

Model evaluation

Key forage and herd outputs of the model were validated by comparing the results from a baseline model simulation with a set of measured data for Wambiana and Northern Queensland. Due to the limited amount of long-term forage and herd measurements available in the literature, we also compared our model outputs with the ones from the GRASP model — model that has been extensively validated for Northern Queensland including Wambiana (e.g. Ash et al., 2015; Mckeon et al., 2000; Scanlan et al., 2013)

Key outputs included temporal variations in forage growth, TSDM and stocking rates as well as mean stocking rates, TSDM, fertility, mortality, calving and weaning rates, forage utilisation rates, forage intake as a function of forage availability, animal liveweight gains and total methane emissions over the relevant time periods.

The model was run including weather data from 1980 to 2011. The weather input came from the weather station of Charters Towers Post Office (01/1980-11/1992, station number 034002) and Airport (12/1992-12/2011, station number 034084), Queensland, Australia (Bureau of Meteorology, 2018). The farming practices were defined as no animal feed supplementation used and a forage type and management corresponding to a mean total standing dry matter of about 2000 kg/ha. The initial stocking rate in 1980 was 0.2 TLU/ha and the initial forage biomass in 1980 was 2000 kg/ha.

The results showed agreement between our model and the evaluation datasets, which gave confidence that the model adequately simulated these production systems (Table 1, Figures 1, 2 and 3).

The relationship between forage availability and animal forage intake captured by the baseline model simulation (Figure 4) was also consistent with the one found in the literature (e.g. Hunt et al. (2014)).

Mean herd utilisation rates (defined as the percentage of annual pasture growth consumed by the herd) were 17%, which is in accordance with conservative herd managements that ensure the sustainability of pasture productivity and land condition in Northern Queensland (Ash et al., 2011; McIvor et al., 1995; Orr et al., 2010).

Considering the high climatic variability profile shown by the last decade records in Wambiana, having the model showing a reasonable agreement with evaluation datasets for such period strengthened the confidence that our model was robust enough to capture variations in forage and herd dynamics under climate variability.

*Table 1: Comparison of key forage and herd outputs of the herd-forage model with evaluation datasets.*

| Variable | Herd-forage model (period) | Evaluation data | Evaluation data details |
| --- | --- | --- | --- |
| Stocking rate (TLU/ha) | 0.24 (1998-2010)  0.24 (1997-2009)  0.22 (1985-2010) | 0.22 | Scanlan et al. (2013) - Moderate stocking rate (Wambiana, yearly data, modelled, 1998-2010) |
|  |  | 0.26  (min: 0.21, max: 0.35) | O’Reagain et al. (2011) – Managements considered: heavy stocking, moderate stocking, variable stocking, rotational wet season spelling coupled with moderate-heavy stocking, Southern Oscillation Index stocking (Wambiana, yearly data, observed, 1997-2009) |
| TSDM (kg/ha) | 1848 (1998-2010) | 2157 | Scanlan et al. (2013) - Moderate stocking rate (Wambiana, yearly data, modelled, 1998-2010) |
|  |  | 1924  (min: 1640, max: 2274) | O’Reagain et al. (2011) – Managements considered: heavy stocking, moderate stocking, variable stocking, rotational wet season spelling coupled with moderate-heavy stocking, Southern Oscillation Index stocking (Wambiana, yearly data, observed, 1998-2010) |
| Annual mortality rate (%) | 4.6 (Herd, 1985-2010)  4.3 (Breeder, 1981-2011) | 3 | Hunt et al. (2014) - Baseline herd mortality rate (Charters Towers, modelled, 1985-2010) |
|  |  | 5 | Scanlan et al. (2013) - Breeder mortality (Wambiana, modelled, 1981-2011, averaged for the five stocking rates reported) |
| Weaning rate (%) | 49 (1985-2010) | 58 | Ash et al. (2015) - Baseline scenario (Northern Queensland, modelled, 1985-2010) |
| Methane (kg CO_2_-eq/ha/yr, GWP=34) | 257 (1985-2010) | 249 | Ash et al. (2015) - Baseline scenario (Northern Queensland, modelled, 1985-2010, 249 for 2901 heads). Published estimate initially expressed based on a Global Warming Potential of 28. |
| Fertility rate | 58 (1998-2010) | 66 (56-74) | McGowan et al. (2014) - Northern Forest region (includes Wambiana) |
| LWG (kg/head/yr) | 135 (1985-2010) | 127 | Ash et al. (2015) - Baseline scenario (Northern Queensland, modelled, 1985-2010) |
|  |  | 137 | Australian National Inventory (Commonwealth of Australia, 2016) - Appendix p.317, seasonal average |
| Forage utilisation rate (%) (forage consumption/forage growth) | 17 (1985-2010) | 27 | Ash et al. (2015) - Baseline scenario (Northern Queensland, modelled, 1985-2010) |


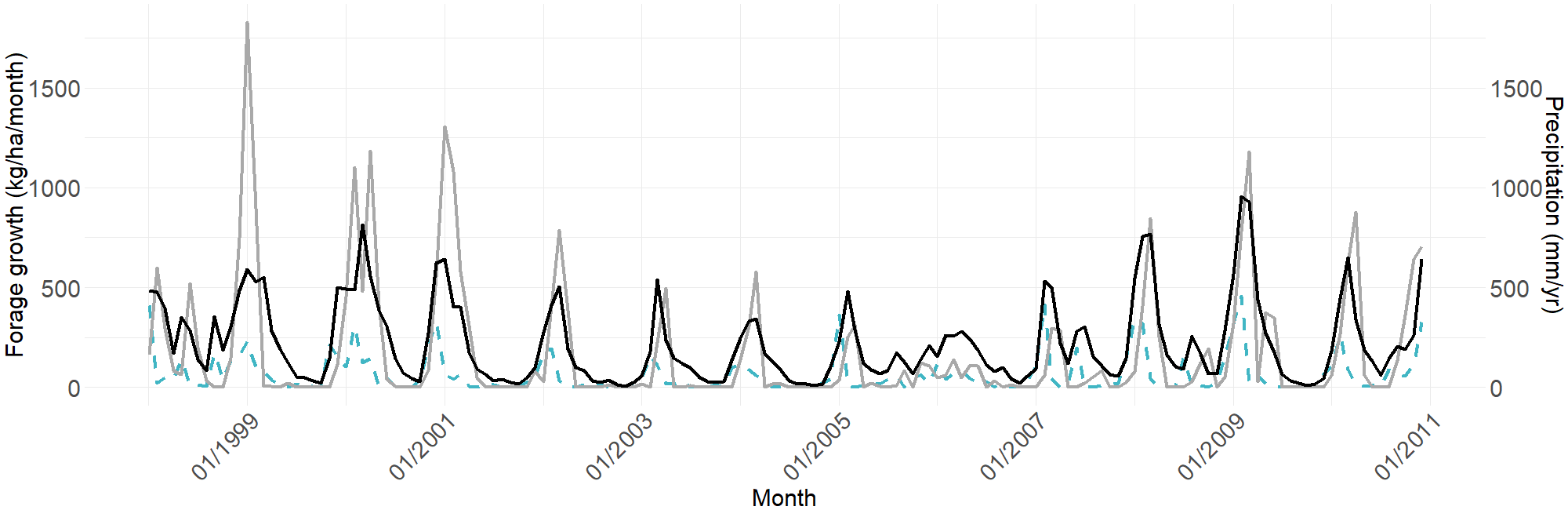


*
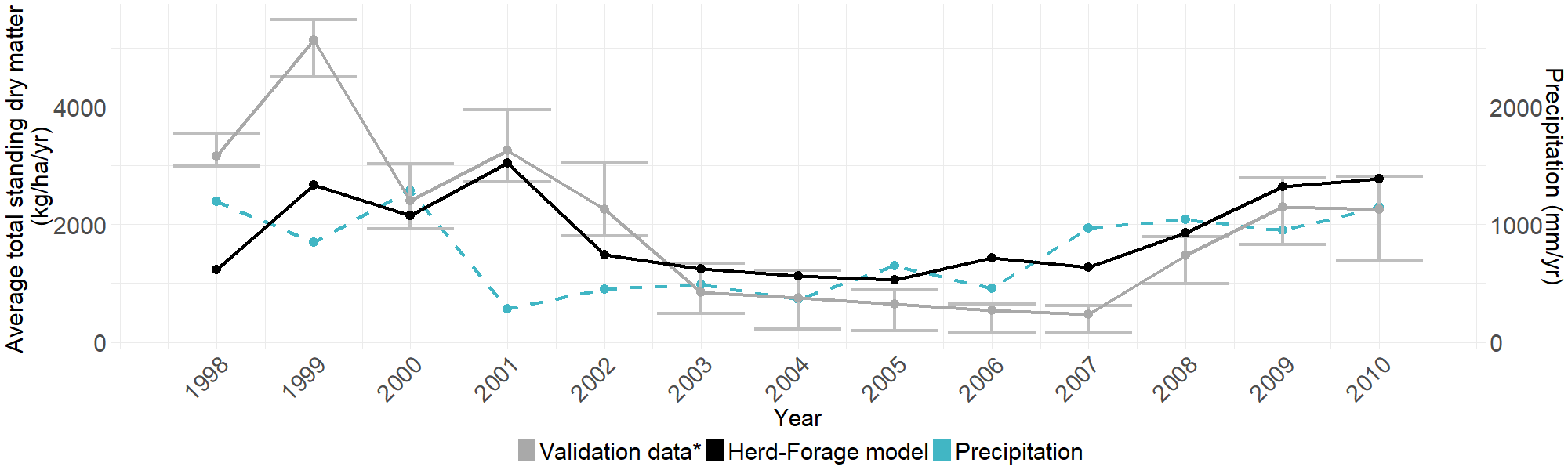
*

*Figure 1: Average monthly forage growth over time predicted by the herd-forage model as compared to an evaluation dataset. Period: 1998-2011. *evaluation data: GRASP model - average of moderate and high stocking rates (Scanlan et al., 2013).*


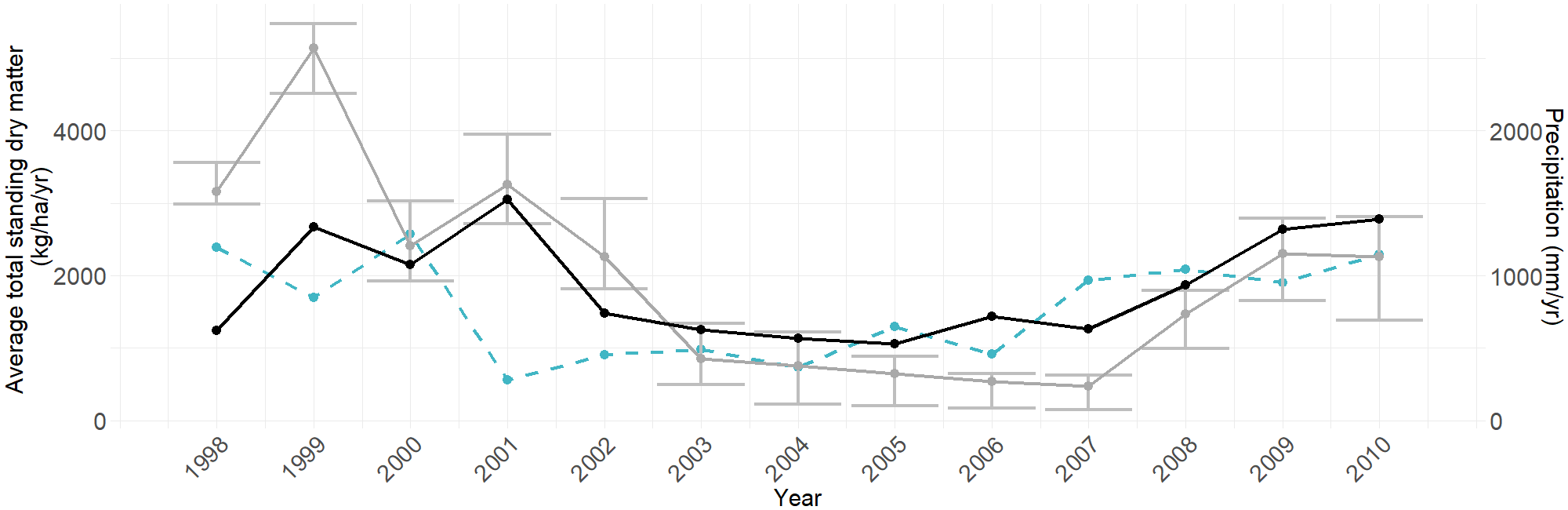


*
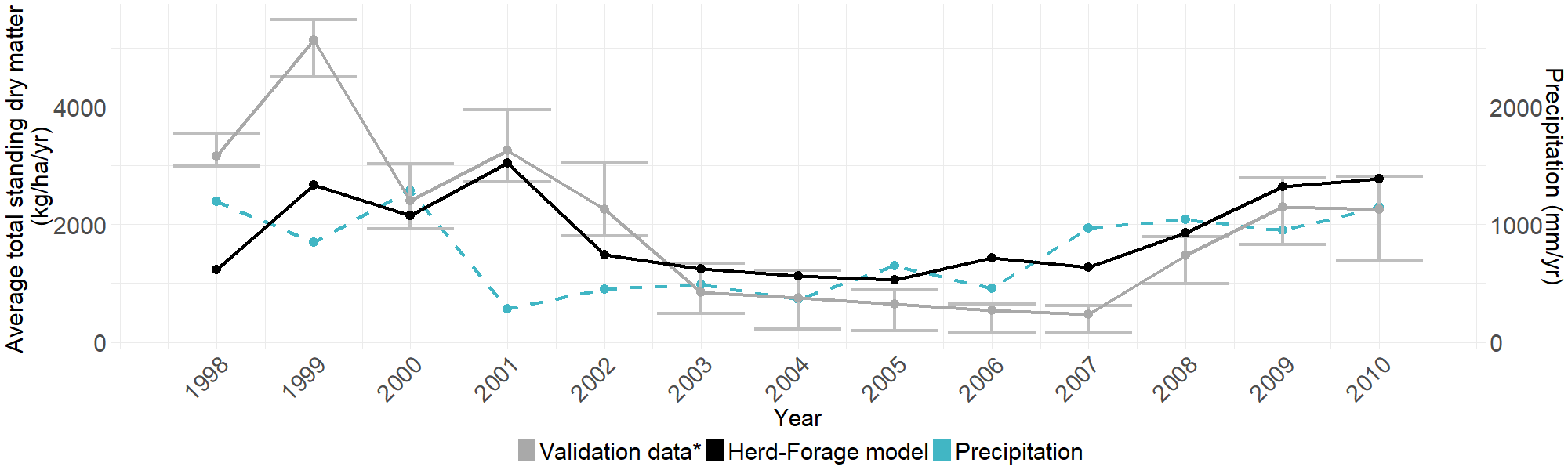
*

*Figure 2: Average annual total standing dry matter over time predicted by the herd-forage model as compared to evaluation datasets. Period: 1998-2010. The error bars represent the minimum and maximum values from the evaluation datasets.*evaluation data: Scanlan et al. (2013) - GRASP model under moderate stocking rate,* O’Reagain et al. (2011) *– heavy stocking, moderate stocking, variable stocking, rotational wet season spelling coupled with moderate-heavy stocking, Southern Oscillation Index stocking.*


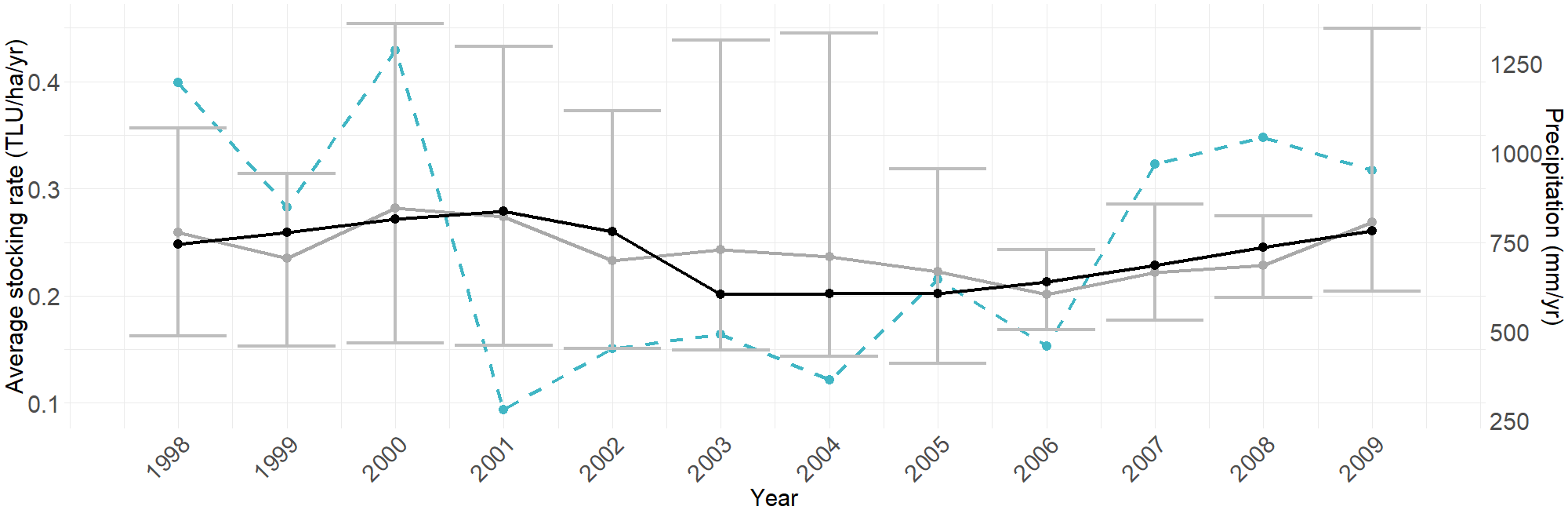


*
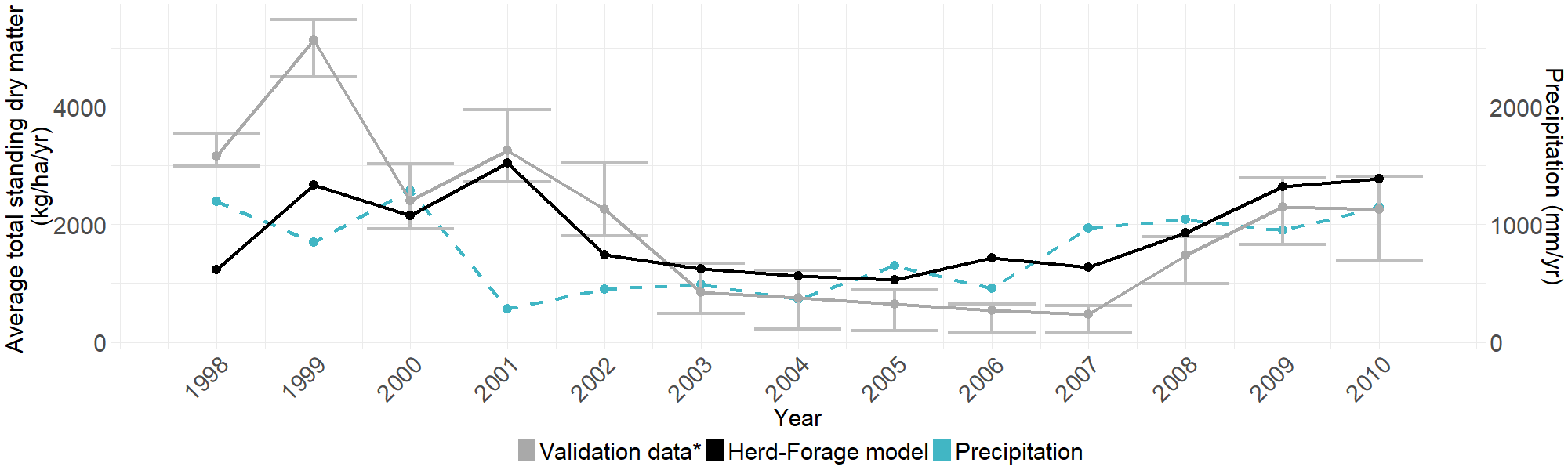
*

*Figure 3: Average annual stocking rates over time predicted by the herd-forage model as compared to evaluation datasets. Period: 1998-2009. The error bars represent the minimum and maximum values from the evaluation datasets.*evaluation data: Ash et al. (2015) –baseline using GRASP model, Scanlan et al. (2013) – GRASP model under moderate stocking rate, O’Reagain et al. (2011) – heavy stocking, moderate stocking, variable stocking, rotational wet season spelling coupled with moderate-heavy stocking, Southern Oscillation Index stocking.*


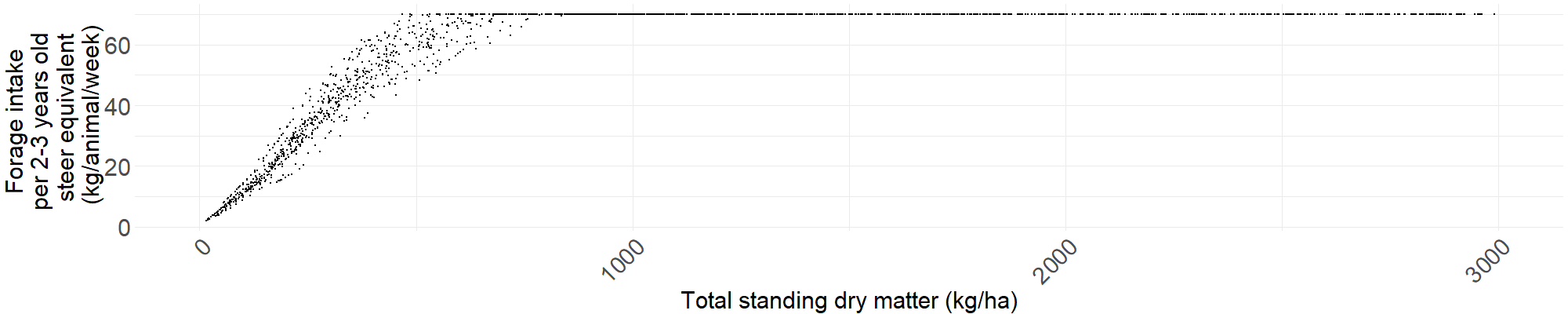


*Figure 4: Relationship between forage availability and animal forage intake as predicted by the herd-forage model.*

In addition to the behaviour tests presented above, we performed other validation tests recommended for system dynamics models including structure assessments (the model structure was built based on an understanding of the structure of the real system), dimension consistency tests (dimensional analysis of the model’s rate equations – variables dimensions indicated in model equations presented further below), extreme condition tests (e.g. under mortality rates equal to 100% or under fertility rates equal to 0%, stocking rates diminish to zero over time, Figure 5) and sensitivity analyses (Senge and Forrester, 1979; Sterman, 2000). Results presented in the main manuscript provide insights as to the sensitivity of forage biomass to different precipitation regimes, precipitation being a key driver influencing forage and consequently herd dynamics. Animals’ birth and death rates are also key variables that influence herd sizes. We present below a sensitivity analysis of the model’s predicted stocking rates to imposed fertility and mortality rates (Figure 5).


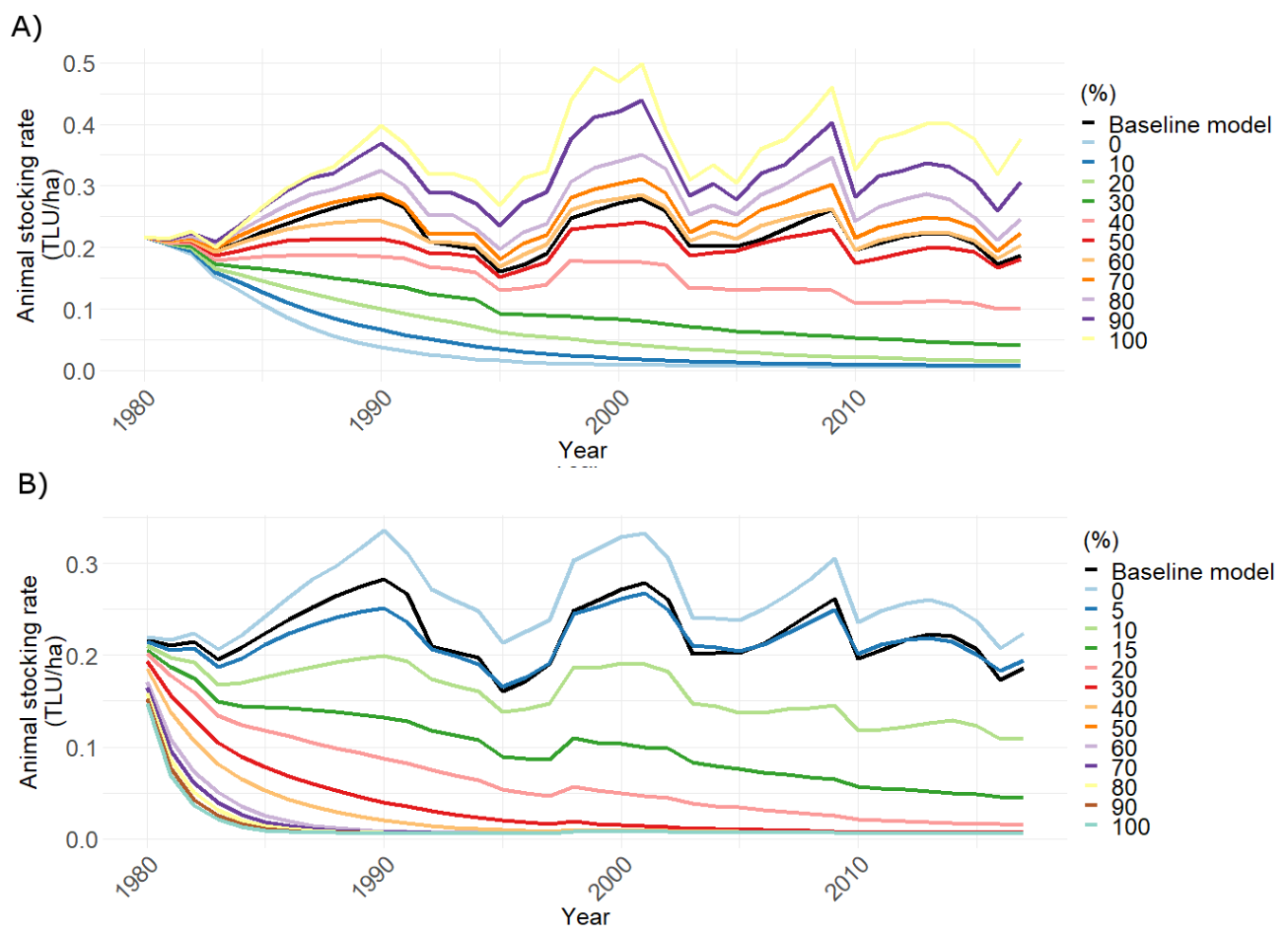


Figure 5: Sensitivity of annual animal stocking rates to (A) breeding cattle fertility rates (%) and (B) adult cattle mortality rates (excl. calves and bulls). In the model, mortality rates are imposed on a weekly basis (here expressed as annual equivalents). Baseline model as described in the main manuscript: fertility and mortality rates depend on forage availability for grazing. Maximum fertility rates equal to 75% and minimum annual adult mortality rates equal to 3%. The initial stocking rate on the 1^st^ of January 1980 was 0.2 TLU/ha and the initial forage biomass was 2000 kg/ha. Due to the system dynamics nature of the model, it takes some time for the imposed fertility and mortality rates to fully propagate through the herd. Weather input from the weather station of Charters Towers Post Office (station number 034002) and Airport (station number 034084), Queensland, Australia (Bureau of Meteorology, 2018).

Model initialisation

A lead-in period of 60 years was used for each climate and farming practices combination to allow the model’s animal stocking rates to stabilise in their spread of values: under very favourable climatic conditions, it takes some years for the stock numbers to increase from their initial stocking rate of 0.2 TLU/ha, as input in the model, to values around 0.6–0.9 TLU/ha through natural replacement with no purchases, as for climate scenario S15. In the first set of climate scenarios (Set 1), the weather input for the lead-in period came from the weather station of Charters Towers Post Office (station number 034002) and Airport (station number 034084), Queensland, Australia (Bureau of Meteorology, 2018). In the second set of climate scenarios (Set 2), the weather input for the lead-in period came from the repetition of the Set 2 30-year long time-series.

Model limitations

Although the herd-forage model was developed to allow the testing of different potential scenarios, the current version of the model was not aimed at capturing the operational diversity and complexities of actual beef enterprises in their entirety. In common with any model of a complex system, it was developed with a specific purpose and is underlined by number of simplifying assumptions, including the following:

- The model represents only a subset of a beef enterprises (e.g. no detailed economic analyses).
- Forage productivity is not influenced by other climate variables than precipitation. It is not influenced by fire regimes neither.
- Forage species composition changes in response to grazing and climate characteristics and thus associated variations in forage quantity and quality are not considered.
- The model doesn’t represent long-term feedbacks of unsustainable forage utilisation rates or droughts on forage productive capacity.
- Changes in water, heat and diseases stress due to climate fluctuations are not represented.
- There is no variation among individuals within a given cohort of animals. All of the animals within a cohort are subject to the same process rates (e.g. grow at the same rate, consume supplement at the same rate).
- The model has no capacity to directly simulate the performance of separate paddocks (we model one single land type in this study), and does not address spatial issues such as uneven grazing distribution and its effect on intake, diet quality and animal production.
- Farming management strategies are simplified

Full list of model equations

This section contains the equations used to create and run the herd and forage model in Stella. The equations are presented in standard iThink/Stella format.

Rainfall and forage type effects on forage productivity

Rainfall = "input weather data"*Effect of change in mean rainfall {mm/week}

Impact of precipitation on growth = GRAPH(Rainfall {1/week})

(0, 0), (50, 187), (100, 375), (150, 480), (200, 500), (250, 500), (300, 500), (350, 500), (400, 500)

Lag time = 6 {weeks}

Lagged impact of precipitation on growth = SMTH1(Impact of precipitation on growth, Lag time, 50) {1/week}

Effect of change in forage species = 1, 1.5 or 2 {unitless}

Forage productivity = Effect of change in forage species*Lagged impact of precipitation on growth {kg/ha/week}

Forage biomass stock

Forage biomass(t) = Forage biomass(t - dt) + (Natural replenishing - Cattle consumption - Senescence Other consumption) * dt

INFLOWS:

Natural replenishing = Total pasture land*Forage productivity {kg/week}

OUTFLOWS:

Cattle consumption = Total Steer 2-3 equivalent*"Actual forage intake per Steer 2-3 eq - step 3" {kg/week}

Rate non farmed cattle consumption Senescence = GRAPH(Senescence counter {1/week})

Senescence counter = COUNTER(1, 53)

Senescence Other consumption = Forage biomass*52*(Rate non farmed cattle consumption Senescence) {kg/week}

Total pasture land = 830 {ha}

Standing forage biomass per ha = (Forage biomass/Total pasture land)*52 {kg/ha/yr}

Effect of annual growing days number on forage quality

Number of annual growing weeks = GRAPH(TIME)

Season counter = COUNTER(1, 53)

season = GRAPH(Season counter)

Additional effect of intra annual variation of rainfall on forage quality = IF Number of annual growing weeks <13 THEN 1 ELSE IF season =3 THEN 1 ELSE 1.2 {rate}

Animal voluntary forage intake

proportion lactating cows = Calves 0-0.5/(Cow 2-3+Cow 3 plus) {unitless}

MA additional intake for milk production = IF proportion lactating cows = 0 THEN 1 ELSE (proportion lactating cows*1.2)+((1-proportion lactating cows)*1) {unitless}

Total Steer 2-3 equivalent = IF season =1 THEN (1/(7.8))*Total voluntary intake ELSE IF season =2 THEN (1/(7.9))*Total voluntary intake ELSE IF season =3 THEN (1/(8.6))*Total voluntary intake ELSE (1/(8.6))*Total voluntary intake {Steer 2-3 equivalent}

Total voluntary intake = IF season =1 THEN (3.1*Weaned male 0.5+3.1*Weaned female 0.5+3.1*Steer 0.5-1+5.8*Steer 1-2+7.8*Steer 2-3+3.1*Cow 0.5-1+5.4*Cow 1-2+7.4*Cow 2-3+7.5*Cow 3 plus+bulls 2-12*9.8)* MA additional intake for milk production ELSE IF season = 2 THEN (3.6*Weaned male 0.5+3.6*Weaned female 0.5+3.6*Steer 0.5-1+6*Steer 1-2+7.9*Steer 2-3+3.6*Cow 0.5-1+5.6*Cow 1-2+7.2*Cow 2-3+7.5*Cow 3 plus+bulls 2-12*9.6)* MA additional intake for milk production ELSE IF season = 3 THEN (4.2*Weaned male 0.5+4*Weaned female 0.5+4.2*Steer 0.5-1+6.7*Steer 1-2+8.6*Steer 2-3+4*Cow 0.5-1+5.8*Cow 1-2+7.4*Cow 2-3+7.6*Cow 3 plus+bulls 2-12*9)* MA additional intake for milk production ELSE (5.3*Weaned male 0.5+4.1*Weaned female 0.5+4.5*Steer 0.5-1+7*Steer 1-2+8.6*Steer 2-3+4.1*Cow 0.5-1+6.1*Cow 1-2+7.1*Cow 2-3+7.5*Cow 3 plus+bulls 2-12*8.6)* MA additional intake for milk production{kg/day}

"Total voluntary intake per TLU – Maintenance" = IF season = 1 THEN ((Total voluntary intake/(Total population TLU per ha*830))*433*7)/250 ELSE IF season = 2 THEN ((Total voluntary intake/(Total population TLU per ha*830))*445*7)/250 ELSE IF season =3 THEN ((Total voluntary intake/(Total population TLU per ha*830))*500*7)/250 ELSE ((Total voluntary intake/(Total population TLU per ha*830))*519*7)/250 {kg/week/Steer 2-3

Animal actual forage intake

Forage biomass available per Steer 2-3 eq = (Forage biomass*52)/Total Steer 2-3 equivalent {kg/Steer 2-3 equivalent}

"Actual forage intake per Steer 2-3 eq - step 1" = GRAPH(Forage biomass available per Steer 2-3 eq {kg/Steer 2-3 equivalent/week})

(1, 0.00), (1300.75, 17.94), (2600.5, 41.92), (3900.25, 58.50), (5200, 70.00)

"Actual forage intake per Steer 2-3 eq - step 2" = IF "Actual forage intake per Steer 2-3 eq - step 1" > "Total voluntary intake per TLU - Maintenance" THEN "Actual forage intake per Steer 2-3 eq - step 1" ELSE IF (Male feed supplementation effect+Female feed supplementation effect)=2 THEN 50 ELSE IF (Male feed supplementation effect+Female feed supplementation effect)=0 THEN "Actual forage intake per Steer 2-3 eq - step 1" ELSE IF Male feed supplementation effect =1 THEN ratio TLU male/female*50+(1-ratio TLU male/female)*"Actual forage intake per Steer 2-3 eq - step 1" ELSE ratio TLU male/female*"Actual forage intake per Steer 2-3 eq - step 1"+(1-ratio TLU male/female)*50 {kg/Steer 2-3 equivalent/week}

"Actual forage intake per Steer 2-3 eq - step 3" = IF "Actual forage intake per Steer 2-3 eq - step 2" <"Total voluntary intake per TLU - Maintenance" THEN "Actual forage intake per Steer 2-3 eq - step 2"* Additional effect of intra annual variation of rainfall on forage quality ELSE "Actual forage intake per Steer 2-3 eq - step 2" {kg/Steer 2-3 equivalent/week}

Average forage intake per Steer 2-3 eq 4weeks = (SMTH3("Actual forage intake per Steer 2-3 eq - step 3", 4))/7 {kg/head/day}

Effect of for intake per Steer 2-3 eq on LWG = GRAPH(Average forage intake per Steer 2-3 eq 4weeks)

(1.00, 0.50), (9.77, 0.52), (18.55, 0.58), (27.33, 0.69), (36.11, 0.84), (44.88, 1.00), (53.66, 1.176), (62.44, 1.47), (71.22, 1.72), (80.00, 2.00)

Effect of cattle forage intake on sales

Average forage intake per Steer 2-3 eq 12 weeks = (SMTH3("Actual forage intake per Steer 2-3 eq - step 3", 12))/7 {kg/head/day}

Proportion male sold when less 4kg per cattle per day = 0.05

Proportion male sold when less 6kg per cattle per day = 0.02

Effect of average forage intake on female sales = IF Average forage intake per Steer 2-3 eq 12 weeks < 2 THEN 0.01 ELSE 0 {1/week}

Effect of average forage intake on male sales = IF Average forage intake per Steer 2-3 eq 12 weeks < 4 THEN proportion male sold when less 4kg per cattle per day ELSE IF Average forage intake per Steer 2-3 eq 12 weeks < 6 THEN proportion male sold when less 6kg per cattle per day ELSE 0 {1/week}

Effect of Average forage intake on sales cow 3 plus= IF Average forage intake per Steer 2-3 eq 12 weeks < 2 THEN 0.02 ELSE 0 {1/week}

Effect of Average forage intake on time to fatten sales Steer 2-3= IF Average forage intake per Steer 2-3 eq 12 weeks < 4 THEN proportion male sold when less 4kg per cattle per day ELSE IF Average forage intake per Steer 2-3 eq 12 weeks < 6 THEN proportion male sold when less 6kg per cattle per day ELSE 0 {1/week}

Emergency Culling female 0.5-1 and 2-3= DELAY(Effect of average forage intake on female sales, 1, 0)*Emergency sales YesNo {1/week}

Emergency Culling male 0.5-3= DELAYN(Effect of average forage intake on male sales, 1, 0)*Emergency sales YesNo {1/week}

Emergency Culling female 3 plus= DELAY(Effect of average forage intake on sales cow 3 plus, 1, 0)*Emergency sales YesNo {1/week}

Emergency Culling Steer 2-3 plus = DELAYN(Effect of Average forage intake on time to fatten sales Steer 2-3 test, 1, 0)*Emergency sales YesNo {1/week}

Emergency sales YesNo = 1 {yes:1, no:0}

Effect of cattle forage intake on fertility

Effect of forage intake per cattle on fertility = GRAPH("Actual forage intake per Steer 2-3 eq - step 1")

(0.00, 0.00), (20.00, 0.18), (40.00, 0.75), (60.00, 0.75), (80.00, 0.75)

Smoothed effect of feed on fertility = SMTH3(Effect of forage intake per cattle on fertility, 16) {unitless}

Effects of cattle forage intake and feeding strategy on mortality

Average forage intake per Steer 2-3 eq 16 weeks = (SMTH3("Actual forage intake per Steer 2-3 eq - step 3", 16))/7 {kg/head/day}

Pulse effect of intake on mortality = IF Average forage intake per Steer 2-3 eq 16 weeks < 4 THEN PULSE( 0.1, 0, 16) ELSE IF Average forage intake per Steer 2-3 eq 16 weeks < 2 THEN PULSE( 0.20, 0, 16) ELSE 0 {1/week}

Smoothed effect of intake on mortality = SMTH3(Pulse effect of intake on mortality, 4)

Minimum female mortality rate = 0.03/52 {1/week}

Minimum male mortality rate = 0.03/52 {1/week}

Non calf female mortality rate = IF Female feed supplementation effect=0 THEN (Minimum female mortality rate+Smoothed effect of intake on mortality) ELSE Minimum female mortality rate {1/week}

Non calf male mortality rate = IF Male feed supplementation effect=0 THEN (Minimum male mortality rate+Smoothed effect of intake on mortality) ELSE Minimum male mortality rate {1/week}

Effect of cattle forage intake on enteric methane emissions

CH4 Charmley eq = ((20.7*Cattle consumption/1000)/Total pasture land) {kgCH4/ha/week}

CH4 in CO2eq Charmley eq = CH4 Charmley eq*34*52 {kgCO2eq/ha/yr}

Land degradation effect and stocking rate adjustment

Natural growth per ha per year = SMTH3(Natural replenishing/830, 52, 50) {kgDM/ha/yr}

Total consumption = Cattle consumption+Senescence Other consumption {kg/week}

Cattle consumption per ha per year = SMTH3(Cattle consumption/830, 52, 13) {kgDM/ha/yr}

Smoothed utilisation rate = Cattle consumption per ha per yr/natural growth per year per ha

Land degradation effect = GRAPH(Smoothed UR)

(0.00, 1.00), (0.10, 1.00), (0.15, 1.00), (0.20, 0.94), (0.30, 0.80), (0.40, 0.68), (0.50, 0.54), (1.00, 0.10)

Desired stocking rate = 0.20*Land degradation effect*Effect of change in forage species {TLU/ha}

Desired number of animals = Total pasture land*Desired stocking rate {TLU}

Stocking rate gap = Total population TLU - Desired number of animals {TLU}

Stocking management decision to reach optimal stocking rate = GRAPH(Stocking rate gap)

(-50.0, 0.00), (-38.88, 0.00), (-27.77, 0.00), (-16.66, 0.00), (0.0, 0.00), (5.55, 0.22), (16.66, 0.34), (27.77, 0.42), (38.88, 0.45), (50.0, 0.45)

Stocking management decision delay = DELAYN(stocking management decision pulse, 6, 20, 0) {1/week}

Stocking management decision pulse = PULSE(Stocking management decision to reach optimal stocking rate, 1, 52) {unitless}

Breeding, gestation and calving

Abortion rate = 0.08 {1/breeding period}

Average litter size = 1 {head/litter}

Breeding duration(t) = Breeding duration(t - dt) + (Start of breeding - End of Breeding) * dt

INIT Breeding duration = 0 {week}

INFLOWS:

Start of breeding = Breeding weeks {1/week}

OUTFLOWS:

End of Breeding = DELAY(Breeding duration, 16, 0) {1/week}

Breeding weeks = PULSE(1, 48, 52) {1/week}

Gestation time = 39 {week}

Gestation(t) = Gestation(t - dt) + (Breeding rate - Birth rate) * dt

INFLOWS:

Breeding rate = IF Female feed supplementation effect=0 THEN (Breeding duration*((Cow 2-3+Cow 3 plus)*Smoothed effect of feed on fertility*Average litter size*Number of deliveries per year)/16) ELSE IF Smoothed effect of feed on fertility <0.5 THEN (Breeding duration*((Cow 2-3+Cow 3 plus)*0.5*Average litter size*Number of deliveries per year)/16) ELSE (Breeding duration*((Cow 2-3+Cow 3 plus)*Smoothed effect of feed on fertility*Average litter size*Number of deliveries per year)/16) {head/week}

OUTFLOWS:

Birth rate = DELAY3(Breeding rate*(1-abortion rate), gestation time, 0.8) {head/week}

Number of deliveries per year = 1 {litter/head/year}

Weaning female 0.5 = DELAY(Birth rate*(1-Calf mortality rate), Weaning time)*(1-Male to female ratio) {head/week}

Calf mortality rate = 0.06/52 {1/week}

Init0= 20 {head}

Calves 0-0.5(t) = Calves 0-0.5(t - dt) + (Birth rate - Weaning male 0.5 - Calves 0-0.5 dying - Weaning female 0.5 - Init0) * dt

INFLOWS:

Birth rate = DELAY3(Breeding rate*(1-abortion rate), gestation time, 0.8) {head/week}

OUTFLOWS:

Calves 0-0.5 dying = Calves 0-0.5*Calf mortality rate {head/week}

Male component

Bulls 2-12(t) = Bulls 2-12(t - dt)

INIT Bulls 2-12 = 5 {head}

Male to female ratio = 0.5 {unitless}

Weaned male 0.5(t) = Weaned male 0.5(t - dt) + (Weaning male 0.5 - Weaned male 0.5 dying - Weaned male 0.5 sold - Becoming Steer 0.5-1) * dt

INIT Weaned male 0.5 = Stk init2 {head}

INFLOWS:

Weaning male 0.5 = DELAY(Birth rate*(1-Calf mortality rate), Weaning time)*Male to female ratio {head/week}

OUTFLOWS:

Weaned male 0.5 dying = Weaned male 0.5*Non calf male mortality rate {head/week}

Weaned male 0.5 sold = Weaned male 0.5*Emergency Culling male 0.5-3{head/week}

Becoming Steer 0.5-1 = Weaned male 0.5/(Time to become Steer 0.5-1) {head/week}

Weaning time = 26 {week}

Steer 0.5-1(t) = Steer 0.5-1(t - dt) + (Becoming Steer 0.5-1 - Becoming Steer 1-2 - Steer 0.5-1 dying - Steer 0.5-1 sold) * dt

INIT Steer 0.5-1 = Stk init4 {head}

INFLOWS:

Becoming Steer 0.5-1 = Weaned male 0.5/(Time to become Steer 0.5-1) {head/week}

OUTFLOWS:

Becoming Steer 1-2 = Steer 0.5-1/(Time to become Steer 1-2) {head/week}

Steer 0.5-1 dying = Steer 0.5-1*Non calf male mortality rate {head/week}

Steer 0.5-1 sold = Steer 0.5-1*Emergency Culling male 0.5-3{head/week}

Steer 1-2(t) = Steer 1-2(t - dt) + (Becoming Steer 1-2 - Becoming Steer 2-3 - Steer 1-2 dying - Steer 1-2 sold) * dt

INIT Steer 1-2 = Stk init5 {head}

INFLOWS:

Becoming Steer 1-2 = Steer 0.5-1/(Time to become Steer 1-2) {head/week}

OUTFLOWS:

Becoming Steer 2-3 = Steer 1-2/(Time to become Steer 2-3) {head/week}

Steer 1-2 dying = Steer 1-2*Non calf male mortality rate {head/week}

Steer 1-2 sold = Steer 1-2*Emergency Culling male 0.5-3{head/week}

Steer 2-3(t) = Steer 2-3(t - dt) + (Becoming Steer 2-3 - Growing out - Steer 2-3 dying) * dt

INIT Steer 2-3 = Stk init6 {head}

INFLOWS:

Becoming Steer 2-3 = Steer 1-2/(Time to become Steer 2-3) {head/week}

OUTFLOWS:

Growing out = IF Emergency sales YesNo = 0 THEN 0 ELSE Steer 2-3/Time to fatten from Steer 2-3 considering forage quality+(Steer 2-3*Emergency Culling Steer 2-3 plus) {head/week}

Steer 2-3 dying = Steer 2-3*Non calf male mortality rate {head/week}

Time to become Steer 0.5-1 = 26*Effect of for intake per Steer 2-3 eq on LWG {week}

Time to become Steer 1-2 = 52*Effect of for intake per Steer 2-3 eq on LWG {week}

Time to become Steer 2-3 = 52*Effect of for intake per Steer 2-3 eq on LWG {weeks}

Time to fatten from Steer 2-3 based on intake per cattle = 52*Effect of for intake per Steer 2-3 eq on LWG {week}

Time to fatten from Steer 2-3 considering forage quality = IF Male feed supplementation effect=0 THEN Time to fatten from Steer 2-3 based on intake per cattle* Additional effect of intra annual variation of rainfall on forage quality ELSE Time to fatten from Steer 2-3 based on intake per cattle {week}

Female component

Weaned female 0.5(t) = Weaned female 0.5(t - dt) + (Weaning female 0.5 - Becoming Cow 0.5-1 - Weaned female 0.5 dying - Weaned female 0.5 sold) * dt

INIT Weaned female 0.5 = Stk init7 {head}

INFLOWS:

OUTFLOWS:

Becoming Cow 0.5-1 = Weaned female 0.5/(Time to become Cow 0.5-1) {head/week}

Weaned female 0.5 dying = Weaned female 0.5*Non calf female mortality rate {head/week}

Weaned female 0.5 sold = Weaned female 0.5*Emergency Culling female 0.5-1 and 2-3{head/week}

Cow 0.5-1(t) = Cow 0.5-1(t - dt) + (Becoming Cow 0.5-1 - Becoming Cow 1-2 - Cow 0.5-1 dying - Cow 0.5-1 sold) * dt

INIT Cow 0.5-1 = Stk init8 {head}

INFLOWS:

Becoming Cow 0.5-1 = Weaned female 0.5/(Time to become Cow 0.5-1) {head/week}

OUTFLOWS:

Becoming Cow 1-2 = Cow 0.5-1/(Time to become Cow 1-2) {head/week}

Cow 0.5-1 dying = Cow 0.5-1*Non calf female mortality rate {head/week}

Cow 0.5-1 sold = Cow 0.5-1*Emergency Culling female 0.5-1 and 2-3{head/week}

Cow 1-2(t) = Cow 1-2(t - dt) + (Becoming Cow 1-2 - Becoming Cow 2-3 - Cow 1-2 dying - Cow 1-2 sold) * dt

INIT Cow 1-2 = Stk init9 {head}

INFLOWS:

Becoming Cow 1-2 = Cow 0.5-1/(Time to become Cow 1-2) {head/week}

OUTFLOWS:

Becoming Cow 2-3 = Cow 1-2/(Time to become Cow 2-3) {head/week}

Cow 1-2 dying = Cow 1-2*Non calf female mortality rate {head/week}

Cow 1-2 sold = IF Emergency sales YesNo = 1 THEN Cow 1-2*stocking management decision delay ELSE 0 {head/week}

Cow 2-3(t) = Cow 2-3(t - dt) + (Becoming Cow 2-3 - Becoming Cow 3 plus - Cow 2-3 dying - Cow 2-3 sold) * dt

INIT Cow 2-3 = Stk init10 {head}

INFLOWS:

Becoming Cow 2-3 = Cow 1-2/(Time to become Cow 2-3) {head/week}

OUTFLOWS:

Becoming Cow 3 plus = Cow 2-3/(Time to become Cow 3 plus) {head/week}

Cow 2-3 dying = Cow 2-3*Non calf female mortality rate {head/week}

Cow 2-3 sold = Cow 2-3*Emergency Culling female 0.5-1 and 2-3{head/week}

Cow 3 plus(t) = Cow 3 plus(t - dt) + (Becoming Cow 3 plus - Cow 3 plus selling to butcher - Cow 3 plus dying) * dt

INIT Cow 3 plus = Stk init11 {head}

INFLOWS:

Becoming Cow 3 plus = Cow 2-3/(Time to become Cow 3 plus) {head/week}

OUTFLOWS:

Cow 3 plus selling to butcher = IF Emergency sales YesNo = 0 THEN 0 ELSE Cow 3 plus /breeding duration from cow 3 plus+(Cow 3 plus*Emergency Culling S C3 test) {head/week}

Cow 3 plus dying = Cow 3 plus*Non calf female mortality rate {head/week}

Time to become Cow 0.5-1 = 26 {week}

Time to become Cow 1-2 = 52 {week}

Time to become Cow 2-3 = 52 {week}

Time to become Cow 3 plus = 52 {week}

Breeding duration from cow 3 plus = 260 {week}

Feed supplementation decision

Female feed supplementation effect = IF Feed supplementation Yes/No =1 THEN 1 ELSE 0 {1: yes, 0: no}

Male feed supplementation effect = IF Feed supplementation Yes/No =0 THEN 0 ELSE IF Female feed supplementation only Yes/No =1 THEN 0 ELSE 1 {1: yes, 0: no}

Feed supplementation Yes/No = 1 {1: yes, 0: no}

Female feed supplementation only Yes/No = 0 {1: yes, 0: no}

Appendix S2 - Climate scenarios characteristics

Set 1 of climate scenarios to study drought period effects on herd crashes and recovery time

*Table 2: Mean annual precipitation, inter and intra-annual coefficients of variation of precipitation of the 13 30-year long climate scenarios input in the herd-forage model. “1 dry year”: one dry year imposed at year 6; “2 dry years”: two consecutive dry years imposed at year 6 and 7, etc. Values highlighted in dark grey are values higher than the third quartile (75^th^ percentile) and those in white are values lower than the first quartile (25^th^ percentile).*

| Climate scenario | Mean precipitation (mm/yr) | Inter-annual coefficient of variation of precipitation, CVP-inter | Intra-annual coefficient of variation of precipitation, CVP-intra |
| --- | --- | --- | --- |
| Baseline | 747 | 0.23 | 0.78 |
| 1 dry year | 729 | 0.26 | 0.76 |
| 1 very dry year | 727 | 0.27 | 0.76 |
| 2 dry years | 714 | 0.28 | 0.77 |
| 2 very dry years | 708 | 0.30 | 0.76 |
| 3 dry years | 704 | 0.29 | 0.76 |
| 3 very dry years | 692 | 0.33 | 0.76 |
| 4 dry years | 693 | 0.31 | 0.78 |
| 4 very dry years | 677 | 0.36 | 0.76 |
| 5 dry years | 679 | 0.33 | 0.80 |
| 5 very dry years | 659 | 0.39 | 0.77 |
| 6 dry years | 670 | 0.34 | 0.81 |
| 6 very dry years | 647 | 0.41 | 0.76 |

*
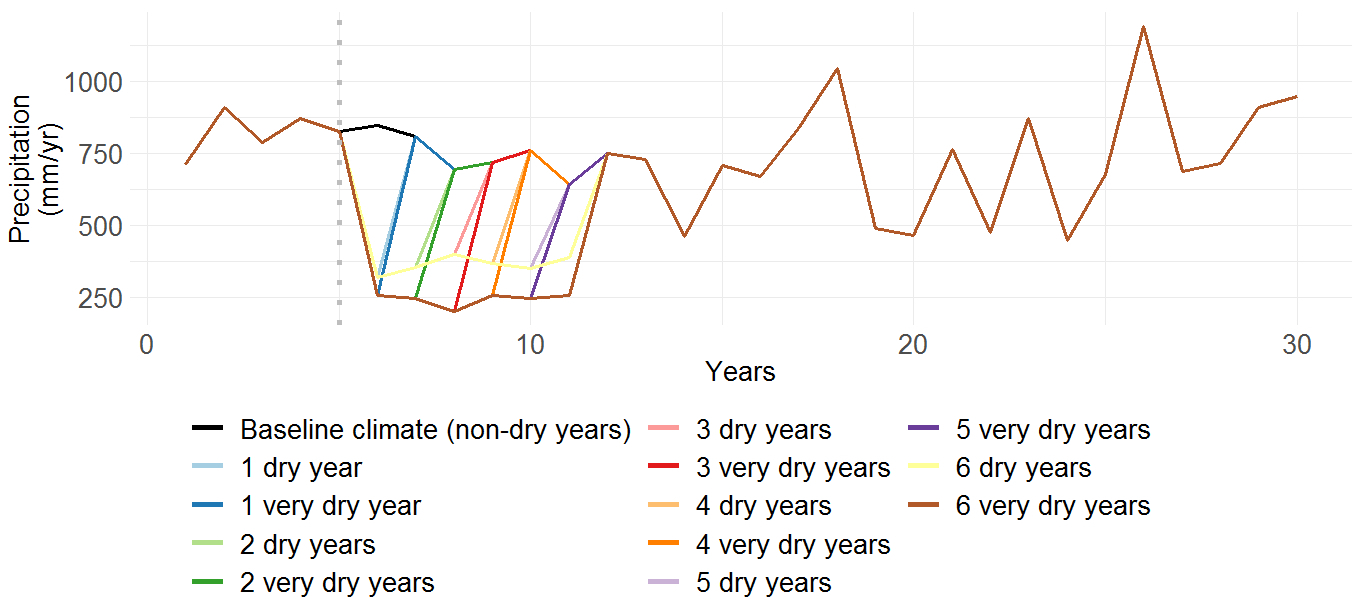
Figure 6: Mean annual precipitation of the 13 30-year long climate scenarios input in the herd-forage model. “1 dry year”: one dry year imposed at year 6; “2 dry years”: two consecutive dry years imposed at year 6 and 7, etc.*

Set 2 of climate scenarios to study the effects of precipitation mean and variability as well as intensification strategies on herd dynamics

*Table 3:* *Mean annual precipitation, inter and intra-annual coefficients of variation of precipitation of the 15 30-year long climate scenarios input in the herd-forage model. Values highlighted in dark grey are values higher than the third quartile (75^th^ percentile) and those in white are values lower than the first quartile (25^th^ percentile). * Characteristics of the historical weather data of Charters Towers Post Office, Queensland, Australia (1900-1992)* (Bureau of Meteorology, 2018)*.*

| Climate scenario | Mean precipitation (mm/yr) | Inter-annual coefficient of variation of precipitation, CVP-inter | Intra-annual coefficient of variation of precipitation, CVP-intra |
| --- | --- | --- | --- |
| S 1 | 751 | 0.29 | 0.86 |
| S 2 | 839 | 0.35 | 1.21 |
| S 3 | 780 | 0.44 | 1.36 |
| S 4 | 712 | 0.27 | 0.51 |
| S 5 | 742 | 0.33 | 0.32 |
| S 6 | 434 | 0.36 | 0.75 |
| S 7 | 433 | 0.39 | 1.21 |
| S 8 | 410 | 0.42 | 1.31 |
| S 9 | 415 | 0.34 | 0.5 |
| S 10 | 373 | 0.35 | 0.24 |
| S 11 | 1054 | 0.28 | 0.81 |
| S 12 | 1157 | 0.31 | 1.22 |
| S 13 | 1087 | 0.35 | 1.38 |
| S 14 | 1067 | 0.29 | 0.43 |
| S 15 | 1091 | 0.28 | 0.27 |
| Average of the 15 climate scenarios | | | |
|  | 756 | 0.34 | 0.83 |
| Precipitation characteristic of the weather data input in Marksim to generate the climate scenarios above* | | | |
| Charters Towers (1900-1992) | 653 | 0.37 | 0.87 |

*
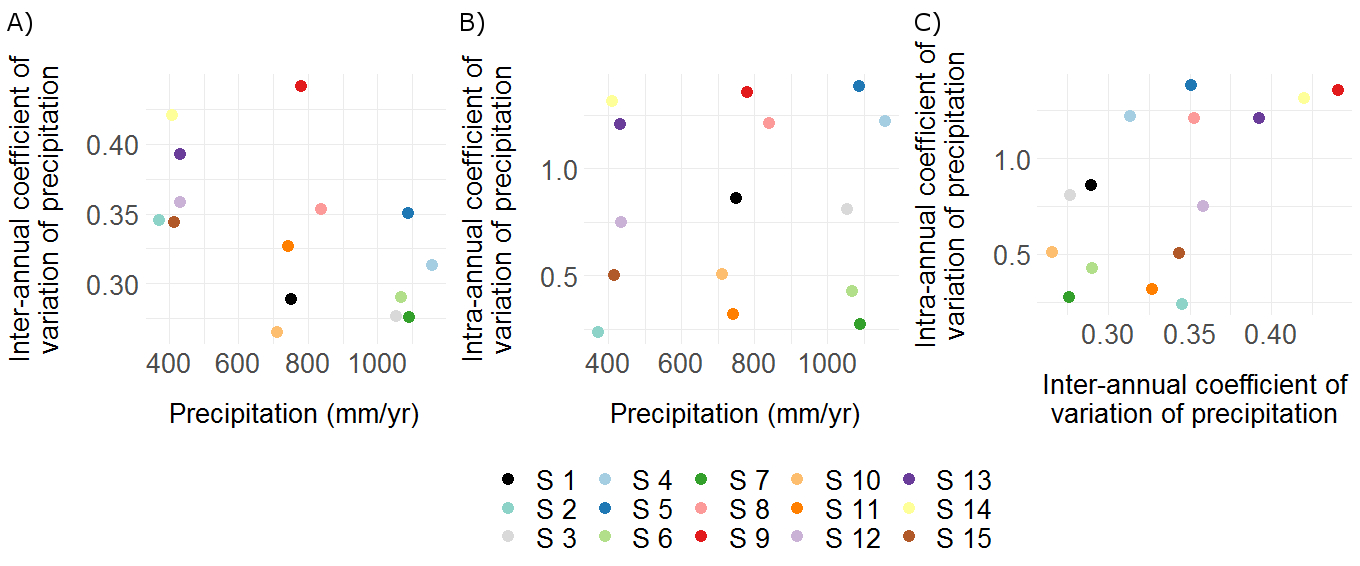
*

*Figure 7: Mean annual precipitation, inter and intra-annual coefficients of variation of precipitation of the 15 30-year long climate scenarios input in the herd-forage model.*

Future climate uncertainties
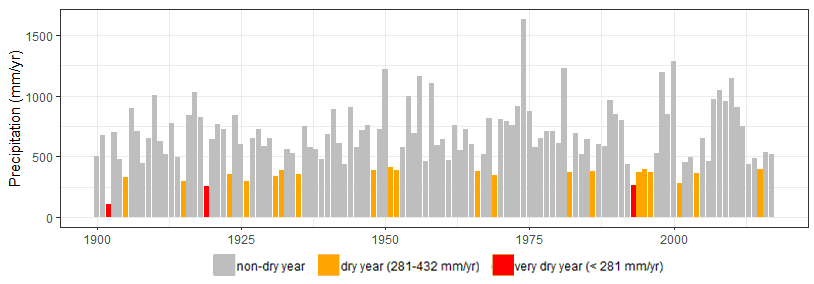


Figure 8: Historical annual rainfall patterns. Wambiana region, Queensland, Australia. Period 1900–2017 (station numbers 34002 (1900–1992) and 34084 (1993–2017), Bureau of Meteorology, 2018).


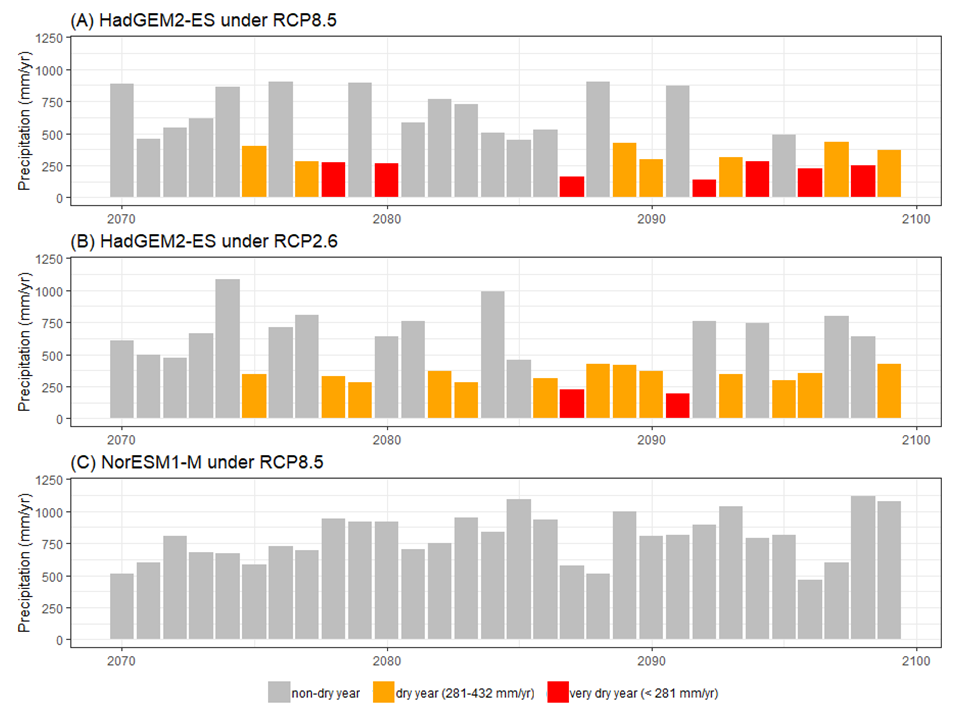


Figure 9: Example of projected annual rainfall patterns from different climate models and Representative Concentration Pathways, which highlight large uncertainties as to climate futures. Wambiana region, Queensland, Australia. Period 2070–2099. Climate scenarios considered: General Circulation Model HadGEM2-ES under Representative Concentration Pathway RCP8.5 (A), HadGEM2-ES under RCP2.6 (B), NorESM1-M under RCP8.5 (C) (Warszawski et al., 2014).

Appendix S3 - Regression tables

Regression tables are presented in the order mentioned in the main manuscript

Under baseline farming management practices

lm(formula = recovery_SR ~ (D_VD + duration), data = df)

Residuals:

Min 1Q Median 3Q Max

-1.5 -0.5 0.0 0.5 1.5

Coefficients:

Estimate Std. Error t value Pr(>|t|)

(Intercept) 8.50000 0.12855 66.122 < 2e-16 ***

D_VDVD 9.00000 0.09717 92.617 < 2e-16 ***

durationl2 1.00000 0.16831 5.941 6.78e-09 ***

durationl3 6.50000 0.16831 38.619 < 2e-16 ***

durationl4 6.50000 0.16831 38.619 < 2e-16 ***

durationl5 5.00000 0.16831 29.707 < 2e-16 ***

durationl6 8.00000 0.16831 47.531 < 2e-16 ***

---

Signif. codes: 0 ‘***’ 0.001 ‘**’ 0.01 ‘*’ 0.05 ‘.’ 0.1 ‘ ’ 1

Residual standard error: 0.9219 on 353 degrees of freedom

(30 observations deleted due to missingness)

Multiple R-squared: 0.9721, Adjusted R-squared: 0.9717

F-statistic: 2053 on 6 and 353 DF, p-value: < 2.2e-16

lm(formula = TSDM ~ CVPinter_ts + CVPintra_ts + mean_precip_ts_mm_yr,

data = df_ts_base)

Residuals:

Min 1Q Median 3Q Max

-99.32 -47.86 -11.73 41.79 123.93

Coefficients:

Estimate Std. Error t value Pr(>|t|)

(Intercept) -57.2083 247.4179 -0.231 0.821

CVPinter_ts -597.4044 685.3675 -0.872 0.402

CVPintra_ts 495.5739 73.5382 6.739 3.20e-05 ***

mean_precip_ts_mm_yr 2.3145 0.1005 23.029 1.17e-10 ***

---

Signif. codes: 0 ‘***’ 0.001 ‘**’ 0.01 ‘*’ 0.05 ‘.’ 0.1 ‘ ’ 1

Residual standard error: 75.46 on 11 degrees of freedom

Multiple R-squared: 0.9917, Adjusted R-squared: 0.9894

F-statistic: 436.8 on 3 and 11 DF, p-value: 1.025e-11

lm(formula = SR ~ CVPinter_ts + CVPintra_ts + mean_precip_ts_mm_yr,

data = df_ts_base)

Residuals:

Min 1Q Median 3Q Max

-0.129093 -0.036371 0.004428 0.053291 0.087592

Coefficients:

Estimate Std. Error t value Pr(>|t|)

(Intercept) 1.879e-01 2.347e-01 0.801 0.440333

CVPinter_ts 2.684e-01 6.501e-01 0.413 0.687687

CVPintra_ts -3.781e-01 6.975e-02 -5.421 0.000210 ***

mean_precip_ts_mm_yr 4.489e-04 9.533e-05 4.709 0.000641 ***

---

Signif. codes: 0 ‘***’ 0.001 ‘**’ 0.01 ‘*’ 0.05 ‘.’ 0.1 ‘ ’ 1

Residual standard error: 0.07157 on 11 degrees of freedom

Multiple R-squared: 0.8956, Adjusted R-squared: 0.8671

F-statistic: 31.46 on 3 and 11 DF, p-value: 1.08e-05

lm(formula = Avg_Total.sold.TLU_ha_yr_ts ~ CVPinter_ts + CVPintra_ts +

mean_precip_ts_mm_yr, data = df_ts_base)

Residuals:

Min 1Q Median 3Q Max

-0.039413 -0.011853 -0.000765 0.017075 0.028105

Coefficients:

Estimate Std. Error t value Pr(>|t|)

(Intercept) 6.526e-02 7.315e-02 0.892 0.391440

CVPinter_ts 5.222e-02 2.026e-01 0.258 0.801393

CVPintra_ts -1.195e-01 2.174e-02 -5.496 0.000187 ***

mean_precip_ts_mm_yr 1.345e-04 2.972e-05 4.526 0.000863 ***

---

Signif. codes: 0 ‘***’ 0.001 ‘**’ 0.01 ‘*’ 0.05 ‘.’ 0.1 ‘ ’ 1

Residual standard error: 0.02231 on 11 degrees of freedom

Multiple R-squared: 0.8988, Adjusted R-squared: 0.8712

F-statistic: 32.55 on 3 and 11 DF, p-value: 9.131e-06

lm(formula = Avg_Total.death.TLU_ha_yr_ts ~ CVPinter_ts + CVPintra_ts +

mean_precip_ts_mm_yr, data = df_ts_base)

Residuals:

Min 1Q Median 3Q Max

-0.0040289 -0.0007764 -0.0000101 0.0011035 0.0037302

Coefficients:

Estimate Std. Error t value Pr(>|t|)

(Intercept) 3.754e-03 7.586e-03 0.495 0.630401

CVPinter_ts 1.687e-02 2.101e-02 0.803 0.439054

CVPintra_ts -1.352e-02 2.255e-03 -5.998 8.95e-05 ***

mean_precip_ts_mm_yr 1.628e-05 3.082e-06 5.283 0.000259 ***

---

Signif. codes: 0 ‘***’ 0.001 ‘**’ 0.01 ‘*’ 0.05 ‘.’ 0.1 ‘ ’ 1

Residual standard error: 0.002314 on 11 degrees of freedom

Multiple R-squared: 0.9062, Adjusted R-squared: 0.8807

F-statistic: 35.44 on 3 and 11 DF, p-value: 6.005e-06

lm(formula = TSDM ~ mean_precip_ts_mm_yr, data = df_ts_base)

Residuals:

Min 1Q Median 3Q Max

-376.09 -166.91 62.86 162.67 253.93

Coefficients:

Estimate Std. Error t value Pr(>|t|)

(Intercept) 57.022 156.377 0.365 0.721

mean_precip_ts_mm_yr 2.439 0.194 12.574 1.19e-08 ***

---

Signif. codes: 0 ‘***’ 0.001 ‘**’ 0.01 ‘*’ 0.05 ‘.’ 0.1 ‘ ’ 1

Residual standard error: 209.7 on 13 degrees of freedom

Multiple R-squared: 0.924, Adjusted R-squared: 0.9182

F-statistic: 158.1 on 1 and 13 DF, p-value: 1.191e-08

lm(formula = SR ~ mean_precip_ts_mm_yr, data = df_ts_base)

Residuals:

Min 1Q Median 3Q Max

-0.22326 -0.11714 -0.01685 0.11600 0.30542

Coefficients:

Estimate Std. Error t value Pr(>|t|)

(Intercept) 0.0243824 0.1271906 0.192 0.8509

mean_precip_ts_mm_yr 0.0003717 0.0001578 2.356 0.0348 *

---

Signif. codes: 0 ‘***’ 0.001 ‘**’ 0.01 ‘*’ 0.05 ‘.’ 0.1 ‘ ’ 1

Residual standard error: 0.1706 on 13 degrees of freedom

Multiple R-squared: 0.2993, Adjusted R-squared: 0.2454

F-statistic: 5.552 on 1 and 13 DF, p-value: 0.03481

lm(formula = SR ~ CVPintra_ts, data = df3_ts_base)

Residuals:

Min 1Q Median 3Q Max

-0.27018 -0.08323 0.02121 0.07232 0.24833

Coefficients:

Estimate Std. Error t value Pr(>|t|)

(Intercept) 0.57760 0.08203 7.041 8.79e-06 ***

CVPintra_ts -0.32965 0.08891 -3.708 0.00263 **

---

Signif. codes: 0 ‘***’ 0.001 ‘**’ 0.01 ‘*’ 0.05 ‘.’ 0.1 ‘ ’ 1

Residual standard error: 0.1421 on 13 degrees of freedom

Multiple R-squared: 0.514, Adjusted R-squared: 0.4766

F-statistic: 13.75 on 1 and 13 DF, p-value: 0.002631

lm(formula = Avg_annual_sales_rate_ts ~ CVPinter_ts + CVPintra_ts +

mean_precip_ts_mm_yr, data = df_ts_base)

Residuals:

Min 1Q Median 3Q Max

-0.019854 -0.005065 0.005394 0.007319 0.010752

Coefficients:

Estimate Std. Error t value Pr(>|t|)

(Intercept) 4.213e-01 3.687e-02 11.427 1.92e-07 ***

CVPinter_ts -3.486e-01 1.021e-01 -3.413 0.005794 **

CVPintra_ts -5.214e-02 1.096e-02 -4.757 0.000593 ***

mean_precip_ts_mm_yr 2.840e-05 1.498e-05 1.896 0.084485 .

---

Signif. codes: 0 ‘***’ 0.001 ‘**’ 0.01 ‘*’ 0.05 ‘.’ 0.1 ‘ ’ 1

Residual standard error: 0.01125 on 11 degrees of freedom

Multiple R-squared: 0.9389, Adjusted R-squared: 0.9222

F-statistic: 56.32 on 3 and 11 DF, p-value: 5.799e-07

lm(formula = Avg_LW.gain.per.head_ts ~ CVPinter_ts + CVPintra_ts +

mean_precip_ts_mm_yr, data = df_ts_base)

Residuals:

Min 1Q Median 3Q Max

-4.5523 -1.3200 0.4695 1.6854 3.5617

Coefficients:

Estimate Std. Error t value Pr(>|t|)

(Intercept) 1.596e+02 8.222e+00 19.406 7.38e-10 ***

CVPinter_ts -8.867e+01 2.278e+01 -3.893 0.00251 **

CVPintra_ts 1.618e+00 2.444e+00 0.662 0.52152

mean_precip_ts_mm_yr -8.025e-04 3.340e-03 -0.240 0.81453

---

Signif. codes: 0 ‘***’ 0.001 ‘**’ 0.01 ‘*’ 0.05 ‘.’ 0.1 ‘ ’ 1

Residual standard error: 2.508 on 11 degrees of freedom

Multiple R-squared: 0.7807, Adjusted R-squared: 0.7209

F-statistic: 13.05 on 3 and 11 DF, p-value: 0.0006051

lm(formula = Avg_annual_death_rate_ts ~ CVPinter_ts + CVPintra_ts +

mean_precip_ts_mm_yr, data = df_ts_base)

Residuals:

Min 1Q Median 3Q Max

-0.0050704 -0.0011853 0.0003874 0.0015806 0.0031663

Coefficients:

Estimate Std. Error t value Pr(>|t|)

(Intercept) 2.784e-02 7.819e-03 3.561 0.00447 **

CVPinter_ts 2.457e-02 2.166e-02 1.134 0.28073

CVPintra_ts -1.854e-03 2.324e-03 -0.798 0.44192

mean_precip_ts_mm_yr 3.843e-06 3.176e-06 1.210 0.25162

---

Signif. codes: 0 ‘***’ 0.001 ‘**’ 0.01 ‘*’ 0.05 ‘.’ 0.1 ‘ ’ 1

Residual standard error: 0.002385 on 11 degrees of freedom

Multiple R-squared: 0.1295, Adjusted R-squared: -0.1079

F-statistic: 0.5457 on 3 and 11 DF, p-value: 0.6612

lm(formula = TSDM ~ mean_precip_ts_mm_yr, data = df_ts_base)

Residuals:

Min 1Q Median 3Q Max

-376.09 -166.91 62.86 162.67 253.93

Coefficients:

Estimate Std. Error t value Pr(>|t|)

(Intercept) 57.022 156.377 0.365 0.721

mean_precip_ts_mm_yr 2.439 0.194 12.574 1.19e-08 ***

---

Signif. codes: 0 ‘***’ 0.001 ‘**’ 0.01 ‘*’ 0.05 ‘.’ 0.1 ‘ ’ 1

Residual standard error: 209.7 on 13 degrees of freedom

Multiple R-squared: 0.924, Adjusted R-squared: 0.9182

F-statistic: 158.1 on 1 and 13 DF, p-value: 1.191e-08

lm(formula = TSDM ~ CVPintra_ts, data = df_ts_base)

Residuals:

Min 1Q Median 3Q Max

-967.27 -719.00 24.15 718.24 996.58

Coefficients:

Estimate Std. Error t value Pr(>|t|)

(Intercept) 1409.5 412.0 3.421 0.00455 **

CVPintra_ts 596.3 446.5 1.335 0.20464

---

Signif. codes: 0 ‘***’ 0.001 ‘**’ 0.01 ‘*’ 0.05 ‘.’ 0.1 ‘ ’ 1

Residual standard error: 713.4 on 13 degrees of freedom

Multiple R-squared: 0.1206, Adjusted R-squared: 0.053

F-statistic: 1.783 on 1 and 13 DF, p-value: 0.2046

lm(formula = TSDM ~ CVPinter_ts, data = df_ts_base)

Residuals:

Min 1Q Median 3Q Max

-1016.9 -515.2 -135.2 475.8 1126.4

Coefficients:

Estimate Std. Error t value Pr(>|t|)

(Intercept) 3454 1228 2.813 0.0147 *

CVPinter_ts -4617 3611 -1.279 0.2234

---

Signif. codes: 0 ‘***’ 0.001 ‘**’ 0.01 ‘*’ 0.05 ‘.’ 0.1 ‘ ’ 1

Residual standard error: 717.1 on 13 degrees of freedom

Multiple R-squared: 0.1117, Adjusted R-squared: 0.04337

F-statistic: 1.635 on 1 and 13 DF, p-value: 0.2234

lm(formula = SR ~ CVPinter_ts, data = df_ts_base)

Residuals:

Min 1Q Median 3Q Max

-0.17676 -0.06104 -0.05033 0.06771 0.25980

Coefficients:

Estimate Std. Error t value Pr(>|t|)

(Intercept) 1.2555 0.2252 5.574 9.01e-05 ***

CVPinter_ts -2.8261 0.6624 -4.267 0.000919 ***

---

Signif. codes: 0 ‘***’ 0.001 ‘**’ 0.01 ‘*’ 0.05 ‘.’ 0.1 ‘ ’ 1

Residual standard error: 0.1315 on 13 degrees of freedom

Multiple R-squared: 0.5834, Adjusted R-squared: 0.5513

F-statistic: 18.2 on 1 and 13 DF, p-value: 0.0009185

lm(formula = SR ~ CVPintra_ts, data = df_ts_base)

Residuals:

Min 1Q Median 3Q Max

-0.27018 -0.08323 0.02121 0.07232 0.24833

Coefficients:

Estimate Std. Error t value Pr(>|t|)

(Intercept) 0.57760 0.08203 7.041 8.79e-06 ***

CVPintra_ts -0.32965 0.08891 -3.708 0.00263 **

---

Signif. codes: 0 ‘***’ 0.001 ‘**’ 0.01 ‘*’ 0.05 ‘.’ 0.1 ‘ ’ 1

Residual standard error: 0.1421 on 13 degrees of freedom

Multiple R-squared: 0.514, Adjusted R-squared: 0.4766

F-statistic: 13.75 on 1 and 13 DF, p-value: 0.002631

lm(formula = SR ~ mean_precip_ts_mm_yr, data = df_ts_base)

Residuals:

Min 1Q Median 3Q Max

-0.22326 -0.11714 -0.01685 0.11600 0.30542

Coefficients:

Estimate Std. Error t value Pr(>|t|)

(Intercept) 0.0243824 0.1271906 0.192 0.8509

mean_precip_ts_mm_yr 0.0003717 0.0001578 2.356 0.0348 *

---

Signif. codes: 0 ‘***’ 0.001 ‘**’ 0.01 ‘*’ 0.05 ‘.’ 0.1 ‘ ’ 1

Residual standard error: 0.1706 on 13 degrees of freedom

Multiple R-squared: 0.2993, Adjusted R-squared: 0.2454

F-statistic: 5.552 on 1 and 13 DF, p-value: 0.03481

lm(formula = CVinter_TSDM_ts ~ CVPinter_ts + CVPintra_ts + mean_precip_ts_mm_yr,

data = df3_ts_base)

Residuals:

Min 1Q Median 3Q Max

-0.046861 0.002023 0.008006 0.009414 0.015079

Coefficients:

Estimate Std. Error t value Pr(>|t|)

(Intercept) 5.094e-02 6.771e-02 0.752 0.467656

CVPinter_ts 1.075e+00 1.876e-01 5.730 0.000132 ***

CVPintra_ts -5.803e-02 2.013e-02 -2.883 0.014885 *

mean_precip_ts_mm_yr -7.939e-06 2.751e-05 -0.289 0.778244

---

Signif. codes: 0 ‘***’ 0.001 ‘**’ 0.01 ‘*’ 0.05 ‘.’ 0.1 ‘ ’ 1

Residual standard error: 0.02065 on 11 degrees of freedom

Multiple R-squared: 0.8729, Adjusted R-squared: 0.8383

F-statistic: 25.19 on 3 and 11 DF, p-value: 3.148e-05

lm(formula = CVinter_SR_ts ~ CVPinter_ts + CVPintra_ts + mean_precip_ts_mm_yr,

data = df3_ts_base)

Residuals:

Min 1Q Median 3Q Max

-0.020668 -0.006387 -0.002564 0.005940 0.018233

Coefficients:

Estimate Std. Error t value Pr(>|t|)

(Intercept) 8.000e-02 3.683e-02 2.172 0.0526 .

CVPinter_ts 7.826e-02 1.020e-01 0.767 0.4592

CVPintra_ts 1.642e-02 1.095e-02 1.500 0.1618

mean_precip_ts_mm_yr -2.650e-05 1.496e-05 -1.771 0.1042

---

Signif. codes: 0 ‘***’ 0.001 ‘**’ 0.01 ‘*’ 0.05 ‘.’ 0.1 ‘ ’ 1

Residual standard error: 0.01123 on 11 degrees of freedom

Multiple R-squared: 0.6484, Adjusted R-squared: 0.5525

F-statistic: 6.762 on 3 and 11 DF, p-value: 0.007532

Intensification scenarios included

lm(formula = TSDM ~ CVPinter_ts + CVPintra_ts + mean_precip_ts_mm_yr +

Effect.of.change.in.forage.species + Energy.Prot.suppl.YesNo,

data = df_ts)

Residuals:

Min 1Q Median 3Q Max

-1429.86 -242.93 31.92 218.56 926.74

Coefficients:

Estimate Std. Error t value Pr(>|t|)

(Intercept) -599.5079 359.6356 -1.667 0.0973 .

CVPinter_ts -2024.7784 987.0803 -2.051 0.0417 *

CVPintra_ts 606.8357 105.9113 5.730 4.38e-08 ***

mean_precip_ts_mm_yr 3.5973 0.1448 24.852 < 2e-16 ***

Effect.of.change.in.forage.species1.5 913.1588 68.7341 13.285 < 2e-16 ***

Effect.of.change.in.forage.species2 1826.4743 68.7341 26.573 < 2e-16 ***

Energy.Prot.suppl.YesNoYes - Energy.Prot.suppl -234.8808 56.1212 -4.185 4.52e-05 ***

---

Signif. codes: 0 ‘***’ 0.001 ‘**’ 0.01 ‘*’ 0.05 ‘.’ 0.1 ‘ ’ 1

Residual standard error: 376.5 on 173 degrees of freedom

Multiple R-squared: 0.9287, Adjusted R-squared: 0.9262

F-statistic: 375.4 on 6 and 173 DF, p-value: < 2.2e-16

lm(formula = Avg_annual_sales_rate_ts ~ CVPinter_ts + CVPintra_ts +

mean_precip_ts_mm_yr + Effect.of.change.in.forage.species +

Energy.Prot.suppl.YesNo, data = df_ts)

Residuals:

Min 1Q Median 3Q Max

-0.039852 -0.003901 0.001458 0.006733 0.019684

Coefficients:

Estimate Std. Error t value Pr(>|t|)

(Intercept) 3.874e-01 9.882e-03 39.205 < 2e-16 ***

CVPinter_ts -2.507e-01 2.712e-02 -9.242 < 2e-16 ***

CVPintra_ts -3.843e-02 2.910e-03 -13.206 < 2e-16 ***

mean_precip_ts_mm_yr 1.560e-05 3.977e-06 3.921 0.000127 ***

Effect.of.change.in.forage.species1.5 7.653e-03 1.889e-03 4.052 7.65e-05 ***

Effect.of.change.in.forage.species2 1.155e-02 1.889e-03 6.115 6.23e-09 ***

Energy.Prot.suppl.YesNoYes - Energy.Prot.suppl 1.786e-02 1.542e-03 11.580 < 2e-16 ***

---

Signif. codes: 0 ‘***’ 0.001 ‘**’ 0.01 ‘*’ 0.05 ‘.’ 0.1 ‘ ’ 1

Residual standard error: 0.01034 on 173 degrees of freedom

Multiple R-squared: 0.8897, Adjusted R-squared: 0.8859

F-statistic: 232.6 on 6 and 173 DF, p-value: < 2.2e-16

lm(formula = Avg_annual_death_rate_ts ~ CVPinter_ts + CVPintra_ts +

mean_precip_ts_mm_yr + Effect.of.change.in.forage.species +

Energy.Prot.suppl.YesNo, data = df_ts)

Residuals:

Min 1Q Median 3Q Max

-0.0054615 -0.0007360 -0.0001679 0.0010285 0.0046705

Coefficients:

Estimate Std. Error t value Pr(>|t|)

(Intercept) 3.241e-02 1.585e-03 20.446 < 2e-16 ***

CVPinter_ts 1.395e-02 4.350e-03 3.207 0.00160 **

CVPintra_ts -1.032e-03 4.668e-04 -2.210 0.02840 *

mean_precip_ts_mm_yr 1.915e-06 6.379e-07 3.001 0.00308 **

Effect.of.change.in.forage.species1.5 9.376e-04 3.029e-04 3.095 0.00229 **

Effect.of.change.in.forage.species2 1.421e-03 3.029e-04 4.690 5.53e-06 ***

Energy.Prot.suppl.YesNoYes - Energy.Prot.suppl -6.925e-03 2.473e-04 -27.997 < 2e-16 ***

---

Signif. codes: 0 ‘***’ 0.001 ‘**’ 0.01 ‘*’ 0.05 ‘.’ 0.1 ‘ ’ 1

Residual standard error: 0.001659 on 173 degrees of freedom

Multiple R-squared: 0.8254, Adjusted R-squared: 0.8194

F-statistic: 136.3 on 6 and 173 DF, p-value: < 2.2e-16

lm(formula = Avg_Total.death.TLU_ha_yr_ts ~ CVPinter_ts + CVPintra_ts +

mean_precip_ts_mm_yr + Effect.of.change.in.forage.species +

Energy.Prot.suppl.YesNo, data = df_ts)

Residuals:

Min 1Q Median 3Q Max

-0.0077685 -0.0026583 -0.0006653 0.0018869 0.0165510

Coefficients:

Estimate Std. Error t value Pr(>|t|)

(Intercept) -1.381e-04 4.092e-03 -0.034 0.97312

CVPinter_ts 2.888e-02 1.123e-02 2.571 0.01098 *

CVPintra_ts -1.745e-02 1.205e-03 -14.485 < 2e-16 ***

mean_precip_ts_mm_yr 2.083e-05 1.647e-06 12.646 < 2e-16 ***

Effect.of.change.in.forage.species1.5 5.106e-03 7.820e-04 6.530 7.05e-10 ***

Effect.of.change.in.forage.species2 1.021e-02 7.820e-04 13.062 < 2e-16 ***

Energy.Prot.suppl.YesNoYes - Energy.Prot.suppl -1.921e-03 6.385e-04 -3.008 0.00302 **

---

Signif. codes: 0 ‘***’ 0.001 ‘**’ 0.01 ‘*’ 0.05 ‘.’ 0.1 ‘ ’ 1

Residual standard error: 0.004283 on 173 degrees of freedom

Multiple R-squared: 0.813, Adjusted R-squared: 0.8065

F-statistic: 125.3 on 6 and 173 DF, p-value: < 2.2e-16

lm(formula = SR ~ CVPinter_ts + CVPintra_ts + mean_precip_ts_mm_yr +

Effect.of.change.in.forage.species + Energy.Prot.suppl.YesNo,

data = df_ts)

Residuals:

Min 1Q Median 3Q Max

-0.24107 -0.08212 -0.01637 0.06094 0.40029

Coefficients:

Estimate Std. Error t value Pr(>|t|)

(Intercept) 1.864e-02 1.243e-01 0.150 0.8810

CVPinter_ts 7.324e-01 3.413e-01 2.146 0.0333 *

CVPintra_ts -5.229e-01 3.662e-02 -14.281 < 2e-16 ***

mean_precip_ts_mm_yr 6.192e-04 5.005e-05 12.373 < 2e-16 ***

Effect.of.change.in.forage.species1.5 1.527e-01 2.376e-02 6.425 1.23e-09 ***

Effect.of.change.in.forage.species2 3.054e-01 2.376e-02 12.852 < 2e-16 ***

Energy.Prot.suppl.YesNoYes - Energy.Prot.suppl 2.522e-02 1.940e-02 1.300 0.1954

---

Signif. codes: 0 ‘***’ 0.001 ‘**’ 0.01 ‘*’ 0.05 ‘.’ 0.1 ‘ ’ 1

Residual standard error: 0.1302 on 173 degrees of freedom

Multiple R-squared: 0.8116, Adjusted R-squared: 0.8051

F-statistic: 124.2 on 6 and 173 DF, p-value: < 2.2e-16

lm(formula = Avg_Total.sold.TLU_ha_yr_ts ~ CVPinter_ts + CVPintra_ts +

mean_precip_ts_mm_yr + Effect.of.change.in.forage.species +

Energy.Prot.suppl.YesNo, data = df_ts)

Residuals:

Min 1Q Median 3Q Max

-0.071479 -0.025539 -0.006837 0.018859 0.123683

Coefficients:

Estimate Std. Error t value Pr(>|t|)

(Intercept) 0.0199991 0.0382678 0.523 0.6019

CVPinter_ts 0.1780956 0.1050323 1.696 0.0918 .

CVPintra_ts -0.1667524 0.0112697 -14.797 < 2e-16 ***

mean_precip_ts_mm_yr 0.0001880 0.0000154 12.204 < 2e-16 ***

Effect.of.change.in.forage.species1.5 0.0456906 0.0073138 6.247 3.14e-09 ***

Effect.of.change.in.forage.species2 0.0913762 0.0073138 12.494 < 2e-16 ***

Energy.Prot.suppl.YesNoYes - Energy.Prot.suppl 0.0092662 0.0059717 1.552 0.1226

---

Signif. codes: 0 ‘***’ 0.001 ‘**’ 0.01 ‘*’ 0.05 ‘.’ 0.1 ‘ ’ 1

Residual standard error: 0.04006 on 173 degrees of freedom

Multiple R-squared: 0.8207, Adjusted R-squared: 0.8145

F-statistic: 132 on 6 and 173 DF, p-value: < 2.2e-16

lm(formula = Avg_LW.gain.per.head_ts ~ CVPinter_ts + CVPintra_ts +

mean_precip_ts_mm_yr + Effect.of.change.in.forage.species +

Energy.Prot.suppl.YesNo, data = df_ts)

Residuals:

Min 1Q Median 3Q Max

-12.5310 -2.2422 0.2452 2.9638 8.6461

Coefficients:

Estimate Std. Error t value Pr(>|t|)

(Intercept) 154.410069 3.604461 42.839 < 2e-16 ***

CVPinter_ts -81.295904 9.893047 -8.217 4.7e-14 ***

CVPintra_ts 0.587819 1.061499 0.554 0.5805

mean_precip_ts_mm_yr 0.003751 0.001451 2.586 0.0105 *

Effect.of.change.in.forage.species1.5 0.272619 0.688890 0.396 0.6928

Effect.of.change.in.forage.species2 0.396710 0.688890 0.576 0.5655

Energy.Prot.suppl.YesNoYes - Energy.Prot.suppl 8.259994 0.562476 14.685 < 2e-16 ***

---

Signif. codes: 0 ‘***’ 0.001 ‘**’ 0.01 ‘*’ 0.05 ‘.’ 0.1 ‘ ’ 1

Residual standard error: 3.773 on 173 degrees of freedom

Multiple R-squared: 0.7397, Adjusted R-squared: 0.7307

F-statistic: 81.95 on 6 and 173 DF, p-value: < 2.2e-16

lm(formula = CVinter_TSDM_ts ~ CVPinter_ts + CVPintra_ts + mean_precip_ts_mm_yr +

Effect.of.change.in.forage.species + Energy.Prot.suppl.YesNo,

data = df_ts)

Residuals:

Min 1Q Median 3Q Max

-0.21244 -0.10780 -0.02135 0.04288 1.07088

Coefficients:

Estimate Std. Error t value Pr(>|t|)

(Intercept) 1.439e-01 2.015e-01 0.714 0.476296

CVPinter_ts 1.143e+00 5.531e-01 2.067 0.040205 *

CVPintra_ts 8.048e-02 5.935e-02 1.356 0.176831

mean_precip_ts_mm_yr -3.139e-04 8.111e-05 -3.870 0.000154 ***

Effect.of.change.in.forage.species1.5 1.352e-03 3.852e-02 0.035 0.972043

Effect.of.change.in.forage.species2 1.643e-03 3.852e-02 0.043 0.966032

Energy.Prot.suppl.YesNoYes - Energy.Prot.suppl 1.918e-01 3.145e-02 6.100 6.74e-09 ***

---

Signif. codes: 0 ‘***’ 0.001 ‘**’ 0.01 ‘*’ 0.05 ‘.’ 0.1 ‘ ’ 1

Residual standard error: 0.211 on 173 degrees of freedom

Multiple R-squared: 0.3998, Adjusted R-squared: 0.3789

F-statistic: 19.2 on 6 and 173 DF, p-value: < 2.2e-16

lm(formula = CVinter_SR_ts ~ CVPinter_ts + CVPintra_ts + mean_precip_ts_mm_yr +

Effect.of.change.in.forage.species + Energy.Prot.suppl.YesNo,

data = df_ts)

Residuals:

Min 1Q Median 3Q Max

-0.023766 -0.012051 -0.000863 0.008138 0.032608

Coefficients:

Estimate Std. Error t value Pr(>|t|)

(Intercept) 1.143e-01 1.410e-02 8.104 9.25e-14 ***

CVPinter_ts -5.506e-03 3.870e-02 -0.142 0.88703

CVPintra_ts 5.915e-04 4.153e-03 0.142 0.88691

mean_precip_ts_mm_yr -1.628e-05 5.676e-06 -2.868 0.00465 **

Effect.of.change.in.forage.species1.5 9.322e-04 2.695e-03 0.346 0.72985

Effect.of.change.in.forage.species2 1.507e-03 2.695e-03 0.559 0.57685

Energy.Prot.suppl.YesNoYes - Energy.Prot.suppl -6.464e-02 2.201e-03 -29.374 < 2e-16 ***

---

Signif. codes: 0 ‘***’ 0.001 ‘**’ 0.01 ‘*’ 0.05 ‘.’ 0.1 ‘ ’ 1

Residual standard error: 0.01476 on 173 degrees of freedom

Multiple R-squared: 0.8356, Adjusted R-squared: 0.8298

F-statistic: 146.5 on 6 and 173 DF, p-value: < 2.2e-16

lm(formula = TSDM ~ Suppl.F.only.YesNo, data = df_ts_feed)

Residuals:

Min 1Q Median 3Q Max

-2057.1 -1156.5 -254.6 925.0 3505.1

Coefficients:

Estimate Std. Error t value Pr(>|t|)

(Intercept) 2548.7 211.5 12.051 <2e-16 ***

Suppl.F.only.YesNoYes - Suppl.F.only.YesNo 142.3 299.1 0.476 0.635

---

Signif. codes: 0 ‘***’ 0.001 ‘**’ 0.01 ‘*’ 0.05 ‘.’ 0.1 ‘ ’ 1

Residual standard error: 1419 on 88 degrees of freedom

Multiple R-squared: 0.002565, Adjusted R-squared: -0.008769

F-statistic: 0.2263 on 1 and 88 DF, p-value: 0.6355

lm(formula = SR ~ Suppl.F.only.YesNo, data = df_ts_feed)

Residuals:

Min 1Q Median 3Q Max

-0.28202 -0.16478 -0.06723 0.09070 0.93945

Coefficients:

Estimate Std. Error t value Pr(>|t|)

(Intercept) 0.46902 0.04009 11.701 <2e-16 ***

Suppl.F.only.YesNoYes - Suppl.F.only.YesNo 0.02102 0.05669 0.371 0.712

---

Signif. codes: 0 ‘***’ 0.001 ‘**’ 0.01 ‘*’ 0.05 ‘.’ 0.1 ‘ ’ 1

Residual standard error: 0.2689 on 88 degrees of freedom

Multiple R-squared: 0.00156, Adjusted R-squared: -0.009786

F-statistic: 0.1375 on 1 and 88 DF, p-value: 0.7117

lm(formula = TSDM ~ mean_precip_ts_mm_yr, data = df_ts)

Residuals:

Min 1Q Median 3Q Max

-1730.62 -589.46 14.23 541.59 1988.82

Coefficients:

Estimate Std. Error t value Pr(>|t|)

(Intercept) -190.3695 186.4727 -1.021 0.309

mean_precip_ts_mm_yr 3.8707 0.2313 16.736 <2e-16 ***

---

Signif. codes: 0 ‘***’ 0.001 ‘**’ 0.01 ‘*’ 0.05 ‘.’ 0.1 ‘ ’ 1

Residual standard error: 866.3 on 178 degrees of freedom

Multiple R-squared: 0.6114, Adjusted R-squared: 0.6092

F-statistic: 280.1 on 1 and 178 DF, p-value: < 2.2e-16

lm(formula = TSDM ~ Effect.of.change.in.forage.species, data = df_ts)

Residuals:

Min 1Q Median 3Q Max

-2667.9 -925.8 143.0 867.0 2626.7

Coefficients:

Estimate Std. Error t value Pr(>|t|)

(Intercept) 1824.1 151.5 12.042 < 2e-16 ***

Effect.of.change.in.forage.species1.5 913.2 214.2 4.263 3.28e-05 ***

Effect.of.change.in.forage.species2 1826.5 214.2 8.526 6.53e-15 ***

---

Signif. codes: 0 ‘***’ 0.001 ‘**’ 0.01 ‘*’ 0.05 ‘.’ 0.1 ‘ ’ 1

Residual standard error: 1173 on 177 degrees of freedom

Multiple R-squared: 0.2911, Adjusted R-squared: 0.2831

F-statistic: 36.35 on 2 and 177 DF, p-value: 5.961e-14

lm(formula = TSDM ~ CVPinter_ts, data = df_ts)

Residuals:

Min 1Q Median 3Q Max

-1922.7 -1000.5 -324.5 761.2 3334.1

Coefficients:

Estimate Std. Error t value Pr(>|t|)

(Intercept) 5786.2 646.9 8.944 4.78e-16 ***

CVPinter_ts -9069.9 1902.5 -4.767 3.87e-06 ***

---

Signif. codes: 0 ‘***’ 0.001 ‘**’ 0.01 ‘*’ 0.05 ‘.’ 0.1 ‘ ’ 1

Residual standard error: 1309 on 178 degrees of freedom

Multiple R-squared: 0.1132, Adjusted R-squared: 0.1082

F-statistic: 22.73 on 1 and 178 DF, p-value: 3.868e-06

lm(formula = TSDM ~ CVPintra_ts, data = df_ts)

Residuals:

Min 1Q Median 3Q Max

-2577.7 -1043.3 -125.7 1111.5 3272.1

Coefficients:

Estimate Std. Error t value Pr(>|t|)

(Intercept) 2175.5 226.8 9.591 < 2e-16 ***

CVPintra_ts 680.7 245.8 2.769 0.00622 **

---

Signif. codes: 0 ‘***’ 0.001 ‘**’ 0.01 ‘*’ 0.05 ‘.’ 0.1 ‘ ’ 1

Residual standard error: 1361 on 178 degrees of freedom

Multiple R-squared: 0.0413, Adjusted R-squared: 0.03591

F-statistic: 7.668 on 1 and 178 DF, p-value: 0.006217

lm(formula = TSDM ~ Energy.Prot.suppl.YesNo, data = df_ts)

Residuals:

Min 1Q Median 3Q Max

-2128.2 -1097.0 -311.1 888.3 3566.3

Coefficients:

Estimate Std. Error t value Pr(>|t|)

(Intercept) 2854.8 146.0 19.559 <2e-16 ***

Energy.Prot.suppl.YesNoYes - Energy.Prot.suppl -234.9 206.4 -1.138 0.257

---

Signif. codes: 0 ‘***’ 0.001 ‘**’ 0.01 ‘*’ 0.05 ‘.’ 0.1 ‘ ’ 1

Residual standard error: 1385 on 178 degrees of freedom

Multiple R-squared: 0.007222, Adjusted R-squared: 0.001644

F-statistic: 1.295 on 1 and 178 DF, p-value: 0.2567

lm(formula = TSDM ~ Suppl.F.only.YesNo, data = df_ts_feed)

Residuals:

Min 1Q Median 3Q Max

-2057.1 -1156.5 -254.6 925.0 3505.1

Coefficients:

Estimate Std. Error t value Pr(>|t|)

(Intercept) 2548.7 211.5 12.051 <2e-16 ***

Suppl.F.only.YesNoYes - Suppl.F.only.YesNo 142.3 299.1 0.476 0.635

---

Signif. codes: 0 ‘***’ 0.001 ‘**’ 0.01 ‘*’ 0.05 ‘.’ 0.1 ‘ ’ 1

Residual standard error: 1419 on 88 degrees of freedom

Multiple R-squared: 0.002565, Adjusted R-squared: -0.008769

F-statistic: 0.2263 on 1 and 88 DF, p-value: 0.6355

lm(formula = SR ~ CVPinter_ts, data = df_ts)

Residuals:

Min 1Q Median 3Q Max

-0.37540 -0.16931 -0.05652 0.10507 0.77828

Coefficients:

Estimate Std. Error t value Pr(>|t|)

(Intercept) 1.6577 0.1149 14.43 <2e-16 ***

CVPinter_ts -3.5424 0.3380 -10.48 <2e-16 ***

---

Signif. codes: 0 ‘***’ 0.001 ‘**’ 0.01 ‘*’ 0.05 ‘.’ 0.1 ‘ ’ 1

Residual standard error: 0.2325 on 178 degrees of freedom

Multiple R-squared: 0.3817, Adjusted R-squared: 0.3782

F-statistic: 109.9 on 1 and 178 DF, p-value: < 2.2e-16

lm(formula = SR ~ CVPintra_ts, data = df_ts)

Residuals:

Min 1Q Median 3Q Max

-0.48980 -0.16452 -0.02081 0.10897 0.75535

Coefficients:

Estimate Std. Error t value Pr(>|t|)

(Intercept) 0.82076 0.03936 20.85 <2e-16 ***

CVPintra_ts -0.42874 0.04266 -10.05 <2e-16 ***

---

Signif. codes: 0 ‘***’ 0.001 ‘**’ 0.01 ‘*’ 0.05 ‘.’ 0.1 ‘ ’ 1

Residual standard error: 0.2361 on 178 degrees of freedom

Multiple R-squared: 0.362, Adjusted R-squared: 0.3584

F-statistic: 101 on 1 and 178 DF, p-value: < 2.2e-16

lm(formula = SR ~ mean_precip_ts_mm_yr, data = df_ts)

Residuals:

Min 1Q Median 3Q Max

-0.42136 -0.19779 -0.03353 0.11325 0.83124

Coefficients:

Estimate Std. Error t value Pr(>|t|)

(Intercept) 1.049e-01 5.668e-02 1.851 0.0658 .

mean_precip_ts_mm_yr 4.786e-04 7.031e-05 6.807 1.47e-10 ***

---

Signif. codes: 0 ‘***’ 0.001 ‘**’ 0.01 ‘*’ 0.05 ‘.’ 0.1 ‘ ’ 1

Residual standard error: 0.2633 on 178 degrees of freedom

Multiple R-squared: 0.2065, Adjusted R-squared: 0.2021

F-statistic: 46.33 on 1 and 178 DF, p-value: 1.471e-10

lm(formula = SR ~ Effect.of.change.in.forage.species, data = df_ts)

Residuals:

Min 1Q Median 3Q Max

-0.4771 -0.1624 -0.1013 0.1472 0.8386

Coefficients:

Estimate Std. Error t value Pr(>|t|)

(Intercept) 0.31422 0.03466 9.066 2.30e-16 ***

Effect.of.change.in.forage.species1.5 0.15269 0.04902 3.115 0.00215 **

Effect.of.change.in.forage.species2 0.30542 0.04902 6.231 3.29e-09 ***

---

Signif. codes: 0 ‘***’ 0.001 ‘**’ 0.01 ‘*’ 0.05 ‘.’ 0.1 ‘ ’ 1

Residual standard error: 0.2685 on 177 degrees of freedom

Multiple R-squared: 0.1799, Adjusted R-squared: 0.1706

F-statistic: 19.41 on 2 and 177 DF, p-value: 2.387e-08

lm(formula = SR ~ Energy.Prot.suppl.YesNo, data = df_ts)

Residuals:

Min 1Q Median 3Q Max

-0.38176 -0.21818 -0.07095 0.11166 1.00396

Coefficients:

Estimate Std. Error t value Pr(>|t|)

(Intercept) 0.45431 0.03113 14.592 <2e-16 ***

Energy.Prot.suppl.YesNoYes - Energy.Prot.suppl 0.02522 0.04403 0.573 0.568

---

Signif. codes: 0 ‘***’ 0.001 ‘**’ 0.01 ‘*’ 0.05 ‘.’ 0.1 ‘ ’ 1

Residual standard error: 0.2954 on 178 degrees of freedom

Multiple R-squared: 0.00184, Adjusted R-squared: -0.003768

F-statistic: 0.328 on 1 and 178 DF, p-value: 0.5675

lm(formula = SR ~ Suppl.F.only.YesNo, data = df_ts_feed)

Residuals:

Min 1Q Median 3Q Max

-0.28202 -0.16478 -0.06723 0.09070 0.93945

Coefficients:

Estimate Std. Error t value Pr(>|t|)

(Intercept) 0.46902 0.04009 11.701 <2e-16 ***

Suppl.F.only.YesNoYes - Suppl.F.only.YesNo 0.02102 0.05669 0.371 0.712

---

Signif. codes: 0 ‘***’ 0.001 ‘**’ 0.01 ‘*’ 0.05 ‘.’ 0.1 ‘ ’ 1

Residual standard error: 0.2689 on 88 degrees of freedom

Multiple R-squared: 0.00156, Adjusted R-squared: -0.009786

F-statistic: 0.1375 on 1 and 88 DF, p-value: 0.7117

Appendix - References

Ash, A., Hunt, L., McDonald, C., Scanlan, J., Bell, L., Cowley, R., Watson, I., McIvor, J., MacLeod, N., 2015. Boosting the productivity and profitability of northern Australian beef enterprises: Exploring innovation options using simulation modelling and systems analysis. Agric. Syst. 139, 50–65. https://doi.org/10.1016/j.agsy.2015.06.001

Ash, A.J., Corfield, J.P., McIvor, J.G., Ksiksi, T.S., 2011. Grazing management in tropical savannas: Utilization and rest strategies to manipulate rangeland condition. Rangel. Ecol. Manag. 64, 223–239. https://doi.org/10.2111/REM-D-09-00111.1

Bureau of Meteorology, 2018. Weather Station Directory [WWW Document]. URL http://www.bom.gov.au/climate/data/stations/ (accessed 8.17.18).

Commonwealth of Australia, 2016. National Inventory Report 2014 (revised) Volume 1.

Hunt, L., Ash, A., MacLeod, N., Mcdonald, C., Scanlan, J., Bell, L., Cowley, R., Watson, I., McIvor, J., 2014. Research opportunities for sustainable productivity improvement in the northern beef industry: A scoping study. Meat & Livestock Australia Limited, North Sydney.

McGowan, M., Fordyce, G., O’Rourke, P., Barnes, T., Morton, J., Sandi, D.M., Jephcott, S., McCosker, K., Smith, D., Perkins, N., Marquart, L., Newsome, T., Burns, B., 2014. Northern Australian beef fertility project: CashCow. Meat & Livestock Australia Limited, North Sydney.

McIvor, J.G., Ash, A.J., Cook, G.D., 1995. Land condition in the tropical tallgrass pasture lands. 1. Effects on herbage production. Rangel. Ecol. Manag. 17, 69–85. https://doi.org/https://doi.org/10.1071/RJ9950069

McKeon, G., Ash, A., Hall, W., Stafford Smith, M., 2000. Simulation of grazing strategies for beef production in north-east Queensland, in: Hammer, G.L., Nicholls, N., Mitchell, C. (Eds.), Applications of Seasonal Climate Forecasting in Agricultural and Natural Ecosystems. Springer, Dordrecht, pp. 227–252. https://doi.org/https://doi.org/10.1007/978-94-015-9351-9_15

O’Reagain, P., Bushell, J., 2011. The Wambiana Grazing Trial: Key Learnings for Sustainable and Profitable Management in a Variable Environment.

Orr, D.M., Burrows, W.H., Hendricksen, R.E., Clem, R.L., Back, P. V., Rutherford, M.T., D. J. Myles, Conway, M.J., 2010. Impacts of grazing management options on pasture and animal productivity in a Heteropogon contortus (black speargrass) pasture in central Queensland. 1. Pasture yield and composition. Crop Pasture Sci. 61, 170–181. https://doi.org/10.1071/AN09145

Scanlan, J.C., Macleod, N.D., O’Reagain, P.J., 2013. Scaling results up from a plot and paddock scale to a property - A case study from a long-term grazing experiment in northern Australia. Rangel. J. 35, 193–200. https://doi.org/10.1071/RJ12084

Senge, P., Forrester, J., 1979. Tests for building confidence in system dynamics models, System dynamics, TIMS studies in management sciences. Cambridge, Massachusetts.

Sterman, J.D., 2000. Business dynamics : systems thinking and modeling for a complex world, Har/Cdr. ed. Boston.

Warszawski, L., Frieler, K., Huber, V., Piontek, F., Serdeczny, O., Schewe, J., 2014. The Inter-Sectoral Impact Model Intercomparison Project (ISI–MIP): Project framework. Proc. Natl. Acad. Sci. 111, 3228–3232. https://doi.org/10.1073/pnas.1312330110
